# ACE2 in chronic disease and COVID-19: gene regulation and post-translational modification

**DOI:** 10.1186/s12929-023-00965-9

**Published:** 2023-08-22

**Authors:** Chia-Wen Wang, Huai-Chia Chuang, Tse-Hua Tan

**Affiliations:** Immunology Research Center, National Health Research Institutes, 35 Keyan Road, Zhunan, 35053 Taiwan

**Keywords:** ACE2, COVID-19, SARS-CoV-2, Chronic disease, Angiotensin II, Transcription, Phosphorylation, Ubiquitination, Glycosylation

## Abstract

Angiotensin-converting enzyme 2 (ACE2), a counter regulator of the renin-angiotensin system, provides protection against several chronic diseases. Besides chronic diseases, ACE2 is the host receptor for SARS-CoV or SARS-CoV-2 virus, mediating the first step of virus infection. ACE2 levels are regulated by transcriptional, post-transcriptional, and post-translational regulation or modification. ACE2 transcription is enhanced by transcription factors including Ikaros, HNFs, GATA6, STAT3 or SIRT1, whereas ACE2 transcription is reduced by the transcription factor Brg1-FoxM1 complex or ERRα. ACE2 levels are also regulated by histone modification or miRNA-induced destabilization. The protein kinase AMPK, CK1α, or MAP4K3 phosphorylates ACE2 protein and induces ACE2 protein levels by decreasing its ubiquitination. The ubiquitination of ACE2 is induced by the E3 ubiquitin ligase MDM2 or UBR4 and decreased by the deubiquitinase UCHL1 or USP50. ACE2 protein levels are also increased by the E3 ligase PIAS4-mediated SUMOylation or the methyltransferase PRMT5-mediated ACE2 methylation, whereas ACE2 protein levels are decreased by AP2-mediated lysosomal degradation. ACE2 is downregulated in several human chronic diseases like diabetes, hypertension, or lung injury. In contrast, SARS-CoV-2 upregulates ACE2 levels, enhancing host cell susceptibility to virus infection. Moreover, soluble ACE2 protein and exosomal ACE2 protein facilitate SARS-CoV-2 infection into host cells. In this review, we summarize the gene regulation and post-translational modification of ACE2 in chronic disease and COVID-19. Understanding the regulation and modification of ACE2 may help to develop prevention or treatment strategies for ACE2-mediated diseases.

## Background

**Fig. 1:**
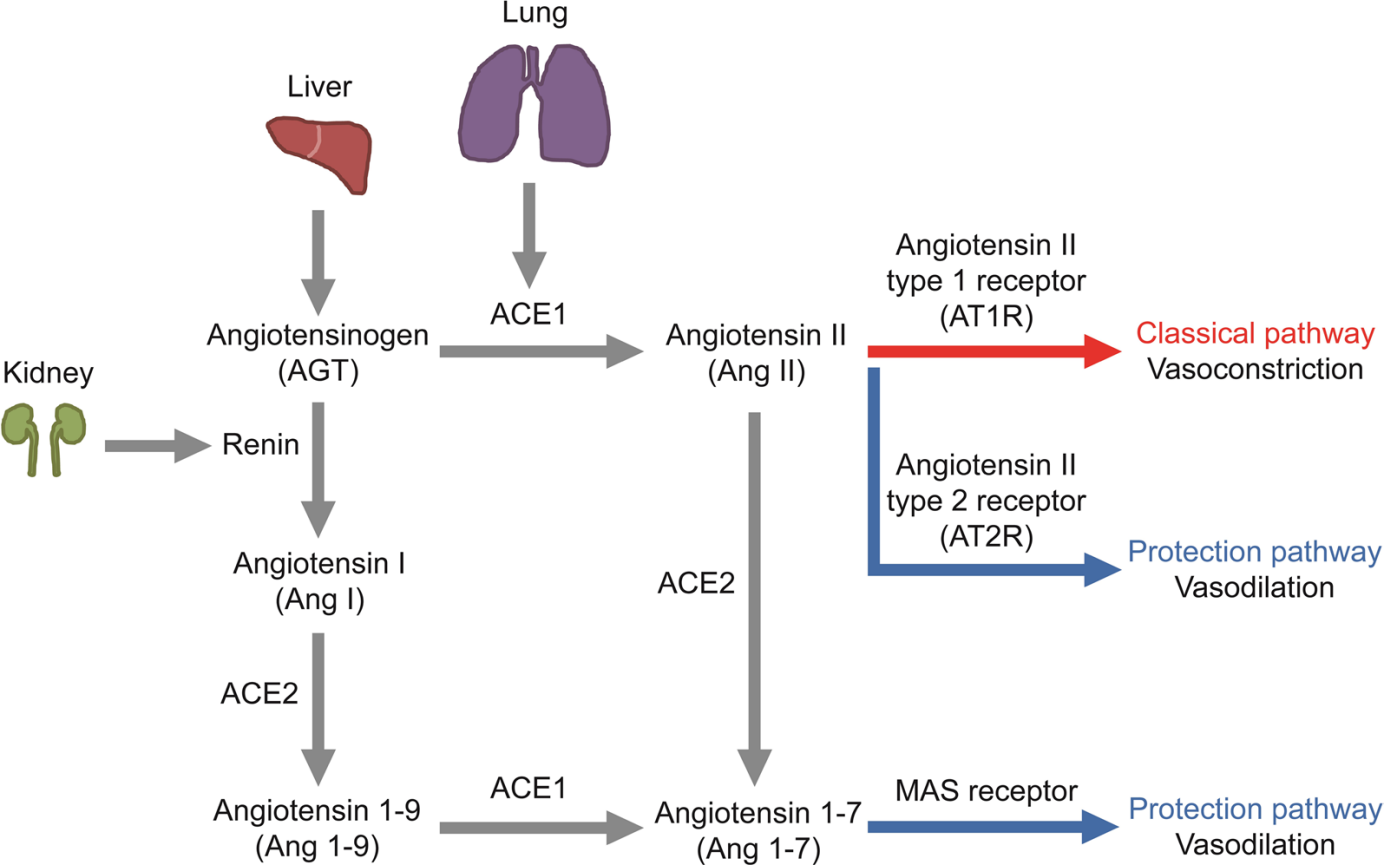
The rennin-angiotensin system (RAS) regulates vasoconstriction and vasodilation. Angiotensin-converting enzyme 1 (ACE1) cleaves angiotensinogen (AGT) into angiotensin II (Ang II), which interacts with Ang II type 1 receptor (AT1R) and leads to vasoconstriction. In contrast, angiotensin-converting enzyme 2 (ACE2) cleaves Ang II into angiotensin 1–7 (Ang 1–7), which binds to the receptor MAS and leads to vasodilation. Moreover, Ang II binds to Ang II type 2 receptor (AT2R) and induces vasodilation

Angiotensin-converting enzyme 2 (ACE2), a homolog of angiotensin-converting enzyme 1 (ACE1), was identified in 2000 [1, 2]. ACE2 is a counter-regulator of the renin-angiotensin system (RAS) (Fig. 1). ACE2 cleaves angiotensin II (Ang II) into angiotensin 1 to 7 (Ang-(1–7)), leading to vasodilation [3]. ACE2 provides protection against several chronic diseases, including cardiovascular diseases, lung injury, and diabetes [4, 5]. Besides chronic diseases, ACE2 plays a crucial role in severe acute respiratory syndrome coronavirus (SARS-CoV) infection and severe acute respiratory syndrome coronavirus 2 (SARS-CoV-2) infection (hereafter named COVID-19). ACE2 is the host receptor for SARS-CoV and SARS-CoV-2 [6, 7]. The attachment of SARS-CoV/SARS-CoV-2 spike (S) protein to ACE2 on the host cell surface is the first step of viral infection [6, 7]. Thus, inhibition of ACE2 may prevent or attenuate the infection of SARA-CoV or SARS-CoV-2.

The ACE2 gene is highly expressed in the thymus, lung, kidney, pancreas, and heart under normal physiological condition according to Gene Expression Omnibus (GEO) and ArrayExpress database [8]. Similar results derived from RNA sequencing and single-cell RNA sequencing (scRNA-seq) show that ACE2 mRNA levels are predominantly expressed in the gastrointestinal tract, kidney, testis, gallbladder, and heart [9]. In addition, ACE2 protein levels are detectable in the kidney, gastrointestinal tract, testis, pancreas, placenta, heart, and gallbladder by mass spectrometry-based proteomics [9]. The ACE2 levels are regulated by age, gender, or pathological stages [10–14]. For example, ACE2 levels are high in the renal cortical tubules and pancreas islets of young diabetic mice [13–20], whereas ACE2 levels are low in the glomerulus and renal tubules of aged diabetic mice [13, 16, 18, 19, 21–24]. In human, ACE2 levels are low in respiratory tract epithelial cells under normal physiological condition [9, 25]. ACE2 mRNA levels in nasal epithelial cells are increased in an age-dependent manner [26]. The mild disease severity of female COVID-19 patients may be due to the reduction of ACE2 levels by the female sex steroid 17β-estradiol in bronchial epithelial cells [12]. Plasma ACE2 concentration is significantly induced in the late stage of severe COVID-19 patients [27]. ACE2 levels in lung epithelial cells are induced by the spike (S) protein of SARS-CoV-2 in human lung diseases [28, 29]. Understanding the modification and regulation of ACE2 would help development of therapeutic strategies against chronic diseases and COVID-19. In this review, we summarize the gene regulation and post-translational modification of ACE2 in chronic disease and COVID-19.

## Regulation of ACE2 gene expression

### Regulation of ACE2 mRNA levels

#### Upregulation of ACE2 transcription

##### Ikaros

**Fig. 2:**
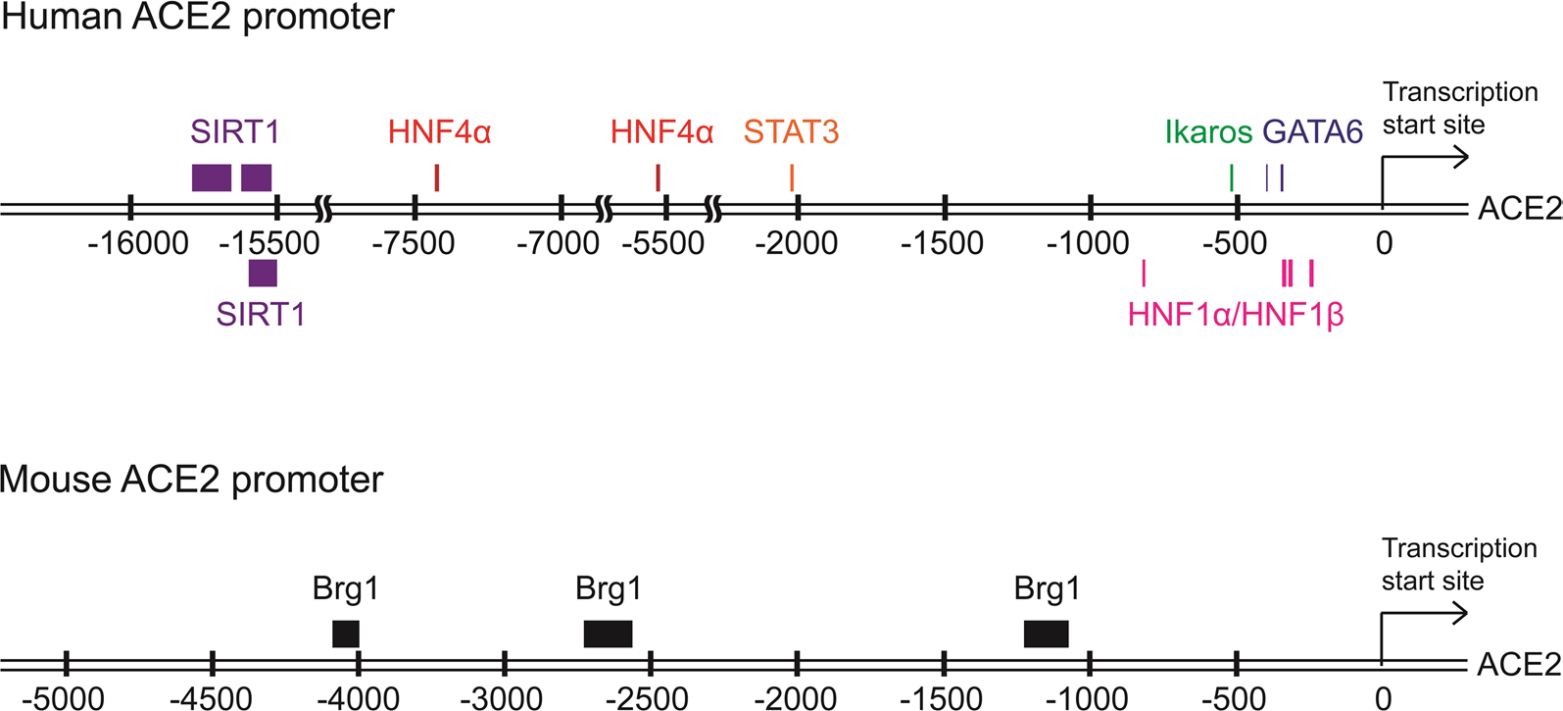
Binding regions of the identified transcription factors in the ACE2 promoter. The numbering of the human and mouse ACE2 promoters are based on Ensembl genome browser (human: ENST00000252519.8; mouse: ENSMUSG00000015405). The numbering for SIRT1 binding sites of the ACE2 promoter are based on UCSC Genome Browser. GATA6: − 351 to − 335, and − 408 to − 403. Ikaros: − 525 to − 519. HNF1α/HNF1β: − 259 to − 242, − 329 to − 312, − 346 to − 330, and − 921 to − 915. STAT3: − 2031 to − 2021. HNF4α: − 5533 to − 5520 and − 7436 to − 7423. SIRT1: − 15,794 to − 15,656, − 15,621 to − 15,521, and − 15,607 to − 15,505. Brg1: − 4089 to − 4001, − 2734 to − 2566, and − 1210 to 1074

ACE2 plays a critical role in the cardioprotection by cleaving angiotensin II (Ang II) [30]. Ang II overexpression stimulates subsequent ACE2 expression, leading to the RAS protection pathway and vasodilation [3]. Ang II treatment induces ACE2 mRNA levels in human cardiofibroblasts [30]. Ikaros (also called Ikaros zinc finger 1, IKZF1) is a hematopoietic zinc finger DNA-binding transcription factor that contributes to the differentiation of human lymphoid and myeloid cells [31]. Ikaros binds to the − 516 to − 481 bp (5′-ATTTGGA-3′) region of the ACE2 promoter in human cardiofibroblasts, promoting ACE2 transcription [30] (Fig. 2, Table 1). The Ang II-induced ACE2 transcription is blocked by mutations of the Ikaros-binding element in the ACE2 promoter [30]. Notably, the Ikaros-mediated ACE2 transcription is not involved in TGF-β or TNF-α signaling [30]. These results indicate that Ikaros binds to the ACE2 promoter at the − 516 to − 481 region and promotes ACE2 transcription in Ang II-stimulated cardiofibroblasts [30].

**Table 1:** The identified transcription factors for the human ACE2 promoter

Transcription factors	Binding regions	Sequence	Ref.
GATA6	− 408 to − 403	TTATCT	[36]
	− 351 to − 341	TCCGTGTATCT	
Ikaros	− 525 to − 519	ATTTGGA	[30]
HNF1α/HNF1β	− 346 to − 330	GTATCTTTAACAGCTTT	[34]
	− 329 to − 312	CTAGGAAAATATTAACCA	
	− 259 to − 242	AGGATTAAAGAATAACGT	
	− 921 to − 915	AGTCATA	[33]
STAT3	− 2031 to − 2021	TTCAACCTTTT	[37]
HNF4α	− 7436 to − 7423	GTGATCTTTGACTC	[35]
	− 5533 to − 5520	ATGTACTTTGCTCT	
SIRT1	− 15,794 to − 15,656	CCCTCCAGAGACGCAGATTACACAACATCCTTCAGTAGTCTGCGTCAATGTTTCAAACTGTGAAGTGATTCTCCCTGAAGACTAAACATGAGGTTTCACTGTGTTCTTTCAGTACATTTCCTCCTGTTCTTTTCTTGCA	[49]
	− 15,621 to − 15,521	TGACGTCAACAAATTTCAAGGCAAAAGTACTCTGTCATTTTCATCTATTTTTTAAAATGATAATTATTTTCTTCTTTAATAACCTTACTAGCTCTTCGGAA	
	− 15,607 to − 15,505	TTCAAGGCAAAAGTACTCTGTCATTTTCATCTATTTTTTAAAATGATAATTATTTTCTTCTTTAATAACCTTACTAGCTCTTCGGAACCTTTACCACATCCCA	

The numbering of the human ACE2 promoter is based on Ensembl genome browser (ID: ENST00000252519.8)

##### HNF1α, HNF1β, and HNF4α

Hepatocyte nuclear factor 1α (HNF1α) and hepatocyte nuclear factor 1β (HNF1β) gene mutations are highly associated with human maturity-onset diabetes of the young (MODY) patients [32]. Interestingly, the ACE2 gene is one of the HNF1β target genes [33]. ACE2 plays a critical role in maintaining normal blood glucose levels and β-cell function [34]. Overexpression of either HNF1α or HNF1β induces ACE2 mRNA levels in mouse pancreatic islet cells and rat insulinoma cells [34]. Both HNF1α or HNF1β bind to the − 346 to − 330 bp (5′-GTATCTTTAACAGCTTT-3′), − 329 to − 312 bp (5′-CTAGGAAAATATTAACCA-3′), and − 259 to − 242 bp (5′-AGGATTAAAGAATAACGT-3′) regions upstream of the ACE2 gene transcriptional start site (Fig. 2, Table 1), resulting in the induction of ACE2 transcription in mouse islet cells and rat insulinoma cells [34]. Moreover, HNF1α and HNF1β bind to the − 818 to − 812 bp region of the ACE2 promoter (Fig. 2, Table 1) and promote ACE2 transcription in human HEK293 embryonic kidney cells [33]. These findings suggest that agonists of HNF1α or HNF1β may be potential targets for MODY by inducing ACE2 levels in pancreatic islet cells. In addition, HNF4α binds to the − 6283 to − 6261 bp (5′-GTGATCTTTGACTC-3′) and − 4380 to − 4358 bp (5′-ATGTACTTTGCTCT-3′) regions of the ACE2 promoter [35] (Fig. 2, Table 1). HNF4α and ACE2 mRNA levels are simultaneously decreased by treatment of the calcineurin inhibitor cyclosporine in human HepG2 cells [35], supporting that HNF4α positively regulates ACE2 transcription.

##### GATA-binding protein 6 (GATA6)

GATA**-**binding protein 6 (GATA6) belongs to a zinc-finger transcription factor family. Genome-wide CRISPR gene knockout screening analysis shows that ACE2 and GATA6 are strongly involved in SARS-CoV-2 infection of human Calu-3 lung epithelial cells [36]. Nasopharyngeal-swab samples from COVID-19 patients and SARS-CoV-2-infected Calu-3 epithelial cells display increased GATA6 mRNA levels [36]. GATA6 knockout Calu-3 epithelial cells are resistant to SARS-CoV-2 infection and infection-induced cell death [36]. Moreover, ACE2 mRNA and protein levels are decreased in GATA6 knockout Calu-3 epithelial cells, suggesting that GATA6 induces ACE2 transcription [36]. GATA6 binds to − 408 to − 403 (5′-TTATCT-3′) and − 351 to − 341 bp (5′-TCCGTGTATCT-3′) regions upstream of the ACE2 gene transcription start site (Fig. 2, Table 1), promoting ACE2 transcription in lung epithelial cells [36]. These results suggest that SARS-CoV-2 enhances ACE2 transcription by inducing GATA6 in human lung epithelial cells, leading to severe SARS-CoV-2 infection [36].

##### Signal transducer and activator of transcription 3 (STAT3)

Tyr705 phosphorylation (activation) signals of signal transducer and activator of transcription 3 (SATA3) are positively correlated with the protein levels of ACE2 in lung tissues from human patients with pulmonary chronic inflammation or lung cancer [37]. STAT3 binds to the ACE2 − 2031 to − 2021 bp (5′-TTCAACCTTTT-3′) region in the ACE2 promoter (Fig. 2, Table 1), promoting the ACE2 transcription in human 16HBE bronchial epithelial cells [37]. Conversely, ACE2 mRNA and protein levels are decreased by STAT3 siRNA knockdown in human 16HBE epithelial cells [37]. Inhibition of IL-6 using a small-molecule compound, 6-*O*-angeloylplenolin, decreases phospho-STAT3 (Tyr705) levels and ACE2 levels in human 16HBE epithelial cells and human Beas-2B bronchial epithelial cells [37]. Moreover, STAT3 activation and ACE2 levels in lung tissues of mice are inhibited by 6-*O*-angeloylplenolin treatment [37]. Besides chronic inflammatory lung tissues, ACE2 protein levels are also increased in synovial tissues of human rheumatoid arthritis patients [38]. IL-6 treatment enhances ACE2 mRNA levels in primary human fibroblast-like synoviocytes [38]; the IL-6-enhanced ACE2 levels are suppressed by STAT3 siRNA knockdown [38]. These findings suggest that IL-6 promotes ACE2 transcription through STAT3 in inflammatory diseases.

##### Silent information regulator T1 (SIRT1)

Silent information regulator T1 (SIRT1), a histone deacetylase, is required for aging, cellular senescence, and energy homeostasis [39, 40]. The AMP-activated protein kinase (AMPK) is a serine/threonine kinase that is activated under energy stress (high AMP to ATP ratio) [41, 42]. The target molecules of AMPK and SIRT1 are highly overlapped [43]. Overexpression of SIRT1 stimulates AMPK phosphorylation and activation [44, 45]. AMPK reciprocally activates SIRT1 [46–48]. ACE2 mRNA levels and SIRT1 protein levels are upregulated by the AMP analogue AICAR (5-amino-4-imidazolecarboxamide riboside, AMPK activator) in human Huh7 hepatoma cells [49]. AICAR induces the binding of SIRT1 to the − 15,794 to − 15,656 bp, − 15,621 to − 15,521 bp, and − 15,607 to − 15,505 bp regions in the ACE2 promoter (Fig. 2, Table 1), enhancing ACE2 transcription in the Huh7 hepatoma cells [49]. The data suggest that ACE2 transcription may be upregulated through the SIRT/AMPK axis.

##### Histone 3

Histone post-translational modifications include acetylation and methylation. Acetylated histone 3 (H3-ac) facilitates DNA dynamics and organization [50, 51]. After feeding high cholesterol diet (HCD), H3-ac and ACE2 protein levels are modestly decreased in the heart tissue of New Zealand white rabbits [52]. In contrast, H3-ac levels and ACE2 mRNA levels are significantly increased by the cholesterol lowering medicine atorvastatin in the heart tissue of HCD-fed rabbits [52]. Atorvastatin stimulates the binding of H3-ac to the ACE2 promoter [52]. The mitigation of atherosclerosis by atorvastatin may be due to the enhancement of ACE2 levels. It would be interesting to study whether the enhancement of ACE2 transcription is mediated by the atorvastatin-induced H3-ac binding.

Besides histone acetylation, histone methylation also regulates gene transcription [53]. ChIP data from Roadmap Epigenomics Project database show that monomethylated histone 3 lysine 4 (H3K4me1), trimethylated histone 3 lysine 4 (H3K4me3), and monoacetylated histone 3 lysine 27 (H3K27ac) bind to the ACE2 locus in the human lung tissue [53]. These findings suggest that epigenetical regulation controls ACE2 levels in human lung tissues [53].

##### Dual specificity tyrosine phosphorylation regulated kinase 1A (DYRK1A)

Dual specificity tyrosine phosphorylation regulated kinase 1A (DYRK1A) modulates cell cycle progression and downregulates neurological development [54, 55]. An extra copy of DYRK1A gene leads to Down syndrome, which shows delayed neurological development [56, 57]. Down syndrome patients show high susceptibility to SARS-CoV-2 virus infection and high hospitalization/death rates [58–61]. DYRK1A knockout decreases ACE2 mRNA and protein levels, leading to attenuation of SARS-CoV-2 infection in monkey Vero E6 kidney cells [62]. Interestingly, both wild-type DYRK1A and kinase-dead mutant increase ACE2 levels in Vero E6 cells, suggesting that DYRK1A-mediated ACE2 upregulation is independent of DYRK1A kinase activity [62]. In contrast, ACE2 levels are decreased by a DYRK1A nuclear-location mutation, indicating that ACE2 levels are induced by the nuclear DYRK1 [62]. ATAC-sequencing data show that DYRK1A promotes chromatin accessibility of the ACE2 promoter and distal enhancer [62]. These results indicate that DYRK1A increases ACE2 mRNA levels by promoting chromatin accessibility, leading to enhancement of SARS-CoV-2 infection [62].

##### Interferons (IFNs): INF-α, IFN-β, and IFN-γ

Interferons (IFNs) are cytokines that are induced during viral infection. IFNs promote the expression of interferon-stimulated genes, which are required for antiviral response [63]. RNA sequencing analysis using nasal airway epithelial cells shows that ACE2 mRNA levels are positively correlated with cytotoxic immune responses and interferon signaling during respiratory virus infection [64]. ACE2 gene expression is induced in the human nasal epithelia and lung tissues after influenza A virus infection [65]. Single-cell RNA sequencing (scRNA-seq) data derived from healthy lung tissues of primates show that ACE2 transcripts co-exist with IFN-α receptor 1 and IFN-γ receptor 2 in type II pneumocytes [65]. ACE2 mRNA levels are increased in IFN-β-stimulated human primary bronchial epithelial cells [8, 66]. Moreover, ACE2 mRNA levels are induced by IFN-α2 and IFN-γ in human Beas-2B bronchial epithelial cells and primary nasal epithelial cells [65]. Interestingly, scRNA-seq data show that ACE2 transcripts are correlated with the interferon-stimulated gene STAT1 transcripts in murine tracheal epithelium, human BEAS-2B bronchial cells, and human primary nasal epithelial cells [65]. Furthermore, chromatin-IP sequencing data show that the ACE2 promoter contains two STAT1/3-binding regions in human cells [65, 67–69]. These results suggest that IFN-α2, IFN-β, and IFN-γ induce ACE2 transcription through STAT1 in virus-infected airway epithelial cells.

##### SMAD4, EP300, PIAS1, and BAMBI

Genome-wide CRISPR gene knockout screening analysis shows that suppressor of mothers against decapentaplegic family member 4 (SMAD4), E1A binding protein P300 (EP300), protein inhibitor of activated STAT1 (PIAS1), and BMP and activin membrane bound inhibitor (BAMBI) positively regulate ACE2 mRNA levels in human Huh7 hepatocytes. In contrast, lysine demethylase 6A (KDM6A) and glycosylphosphatidylinositol anchor attachment 1 (GPAA1) decrease ACE2 protein levels in Huh7 hepatocytes [70]. However, whether ACE2 mRNA levels are inhibited by KDM6A or GPAA1 is unclear.

#### Downregulation of ACE2 transcription

##### Brg1-FoxM1 complex

ACE2 mRNA levels are decreased in the heart tissues of transaortic constriction (TAC)-induced heart failure mice [71]. In contrast, mRNA and protein levels of both brahma-related gene-1 (Brg1, also named SMARCA4) and forkhead box M1 (FoxM1) are upregulated in TAC treated-mouse cardiomyocytes and endothelial cells [71]. Brg1 is a chromatin-remodeling ATPase; FoxM1 is a transcription factor. The upregulation of Brg1 is positively correlated with the disease development of human hypertrophic cardiomyopathy [72]; conversely, the cardiac hypertrophy is reduced in Brg1-deficient mice [71]. TAC-reduced ACE2 protein levels in the mouse heart endothelial cells are recovered by Brg1 deficiency or FoxM1 inhibition [71]. Chromatin immunoprecipitation (ChIP) data show that Brg1-binding sites are located in the mouse ACE2 promoter at − 4089 to − 4001 bp, − 2734 to − 2566 bp, and − 1210 to 1074 bp, which are highly homologous to human and rat [71]. Overexpression of Brg1 in mouse cardiac endothelial cells causes a reduction of ACE2 promoter activity [71]. Results of co-immunoprecipitation and proximity ligation assay indicate that Brg1 protein and FoxM1 protein form a complex, which controls ACE2 transcription in mouse cardiac endothelial cells [71]. FoxM1 induces Brg1-mediated ACE2 transcriptional downregulation [71]. These results indicate that the Brg1-FoxM1 protein complex binds to the ACE2 promoter, leading to inhibition of ACE2 transcription [71].

##### Estrogen-related receptor α (ERRα)

The transcription factor, estrogen-related receptor α (ERRα, also named NR3B1) belongs to the nuclear receptor superfamily. ERRα protein is expressed in the human heart, liver, kidney, and brown adipose tissues [73]. ERRα binds to the ACE2 promoter, inhibiting ACE2 transcription in the kidney of C57/Bl6 mice [74]. ACE2 levels are increased in ERRα KO mice during the dark phase of the zeitgeber time compared to wild-type (WT) mice, leading to lower kidney blood pressure [74]. These results suggest that ERRα suppresses ACE2 transcription by binding to the ACE2 promoter, leading to higher blood pressure in the kidney [74].

##### Zeste homologue 2 (EZH2)

Histone methylation controls gene expression levels by promoting [53] or suppressing [75, 76] transcription. Zeste homologue 2 (EZH2) is a di/trimethylase that methylates histone 3, resulting trimethylation of histone 3 on lysine 27 (H3K27me3), a marker for repressed enhancer [76]. ACE2 mRNA levels are induced by H3K27 mutation or EZH2 knockout in mouse embryonic stem cells [77]. Moreover, ChIP-seq data show that the binding of H3K27me3 to the ACE2 promoter is reduced by EZH2 knockout in mouse or human ESCs [77]. In contrast, EZH2 knockout increases the binding of monoacetylated histone 3 on lysine 27 (H3K27ac), a marker for active enhancer, to the ACE2 promoter in human ESCs [77]. These results indicate that EZH2 induces H3K27me3 binding to the ACE2 promoter, leading to downregulation of ACE2 gene expression [77].

#### Downregulation of ACE2 mRNA by miRNAs

##### miR-125b

**Fig. 3:**
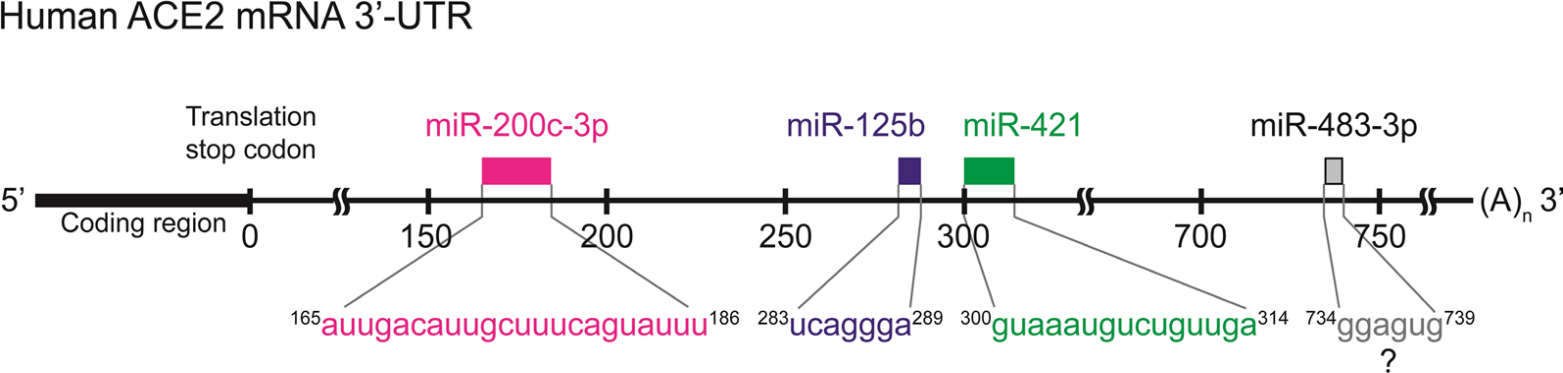
Binding regions of the identified miRNAs in the ACE2 3′-UTR. The numbering of the human ACE2 3′-UTR is based on NCBI nucleotide database (NM_001371415). miR-200c-3p: 165 to 186. miR-125b: 283 to 289. miR-421: 300 to 314. miR-483-3p putative binding site: 734 to 739. The miRNA-targeted sequences of the ACE2 3′-UTR are showed at the bottom

Hyperglycemia of type 1 or type 2 diabetes patients induces renal tubular injury, leading to diabetic nephropathy in the late stage of diabetic renal disease [78]. ACE2 mRNA and protein levels are decreased in the kidney of human type 1 or type 2 diabetes patients [21]; decreased ACE2 levels are correlated with severity of diabetic nephropathy [79]. Enhancement of ACE2 levels attenuates diabetic nephropathy [80]. There are several microRNA-binding sites in the 3′-UTR of ACE2 [79, 81]. High glucose exposure increases microRNA 125b (miR-125b) levels in human HK2 kidney tubular epithelial cells [79]. Conversely, ACE2 protein levels are decreased in high glucose-treated human HK2 epithelial cells [79]. miR-125b binds to the 283 to 289 bp (5′-ucaggga-3′) region in the ACE2 3′-UTR, leading to reduction of ACE2 mRNA stability in HK2 epithelial cells under high-glucose condition [79] (Fig. 3). The high-glucose-decreased ACE2 levels in human HK2 epithelial cells are recovered by inhibition of miR-125b using anti-miR oligonucleotides [79]. Moreover, overexpression of miR-125b induces reactive oxygen species (ROS) formation and apoptosis response in human HK-2 epithelial cells under normal glucose condition [79]. Conversely, high-glucose-induced ROS formation and apoptosis response are obliterated by miR-125b knockout [79]. Deletion of miR-125b target site in the ACE2 3′-UTR blocks the induction of ROS formation and apoptosis response in high glucose-treated HK2 epithelial cells [79]. These findings suggest that high-concentration glucose causes ACE2 downregulation by miR-125b, leading to enhancement of cell apoptosis in kidney tubular epithelial cells [79]. Thus, inhibition of miR-125b may be a potential therapeutic strategy for diabetic nephropathy.

##### miR-200c-3p

ACE2 levels are reduced in H5N1 influenza virus-infected human A549 lung adenocarcinoma cells and patients with pathogen-induced acute respiratory distress syndrome or acute lung injury [82]. ACE2 deficiency increases the lung injury in the H7N9 influenza virus-infected mice [83]. The expression levels of miR-200c-3p (microRNA 200c from the 3′ end of precursor microRNA hairpin) are induced by H5N1 influenza virus infection in A549 cells [82]. The H5N1 influenza virus-stimulated miR-200c-3p induction is blocked by NF-κB inhibition [82]. Overexpression of miR-200c-3p-mimicking miRNA downregulates the reporter activity of the ACE2 3′-UTR [82]. Deletion of the miR-200c-3p binding site in the ACE2 3′-UTR (165 to 186 bp, 5′-auugacauugcuuucaguauuu-3′) results in the enhancement of ACE2 3′-UTR reporter activity [82] (Fig. 3). Collectively, NF-κB-induced miR-200c-3p binds to the ACE2 3′-UTR and inhibits ACE2 mRNA stability in influenza virus-infected lung cells. Furthermore, inhibition of miR-200c-3p by antagomiRNA results in enhancement of ACE2 levels in the lung tissues of H5N1-infected mice, protecting them from virus-induced mortality [82]. Thus, miR-200c-3p may be a potential therapeutic target for human virus-induced pneumonia [82].

##### miR-421

The ACE2 3′-UTR contains a target site (300 bp to 314 bp, 5′-guaaaugucuguuga-3′) of microRNA 421 (miR-421) (Fig. 3). Overexpression of miR-421 decreases the reporter activity of ACE2 3′-UTR by 30% in human Huh7 hepatoma cells [81]. Moreover, miR-421 overexpression results in a 30% decrease of ACE2 protein levels in human primary cardiac myofibroblasts [81]. Mutation of miR-421 binding site within the ACE2 3′-UTR reverses ACE2 protein levels in Huh7 cells [81]. These results suggest that miR-421 is also a miRNA that downregulates ACE2 levels [81].

##### miR-483-3p

microRNA library screening using rat aortic smooth muscle cells (RASMC), human aortic smooth muscle cells (HASMC), mouse atrial cardiomyocytes, and human HEK293 embryonic kidney cells reveals 22 vascular smooth muscle cell (VSMC)-enriched miRNAs that are regulated by Ang II treatment [84]. Among these 22 microRNAs, miR-483-3p (microRNA 483 from the 3′ end of precursor microRNA hairpin) may target and inhibit the 3′-UTR of four Rennin-angiotensin system (RAS) components, including angiotensinogen (AGT), ACE1, ACE2, and the Ang II type 2 receptor (AT2R) [84]. Overexpression of miR-483-3p inhibits the protein levels of AGT and ACE1 in RASMC cells [84]. The ACE2 3′-UTR contains a putative binding element (734 bp to 739 bp, 5′-ggagug-3′) for miR-483-3p [84] (Fig. 3); however, the direct effect of miR-483-3p on ACE2 or AT2R protein levels has not been demonstrated. As miR483-3p may downregulate ACE2 and AT2R levels, miR-483-3p would not be a therapeutic target for cardiovascular diseases due to the opposing functions of AGT/ACE1 versus ACE2/AT2R.

#### Other activators of ACE2 mRNA levels

##### Apelin

Apelin (also known as APLN) is an endogenous peptide that interacts with the G-protein-coupled apelin receptor (APLNR, also called APJ), which is a homolog of the Ang II type 1 receptor (AT1R) [85]. ACE2 mRNA levels are decreased in the heart tissues of heart failure mice [71]. Active apelin enhances ACE2 transcription in apelin receptor (APJ)-overexpressing cardiomyocytes [85]. In contrast, apelin knockout mice display decreased ACE2 levels in the heart tissues and develop severe heart failure [85]. The decrease of ACE2 levels in apelin-deficient heart tissues is rescued by treatment of the AT1R blocker losartan [85]. Furthermore, transaortic constriction-induced cardiac hypertrophy in mice is attenuated by treatment of active apelin [85]; the symptoms attenuated by apelin may be due to its induction of ACE2 levels.

##### Elabela (ELA)

Elabela (ELA) is an endogenous peptide ligand of apelin receptor (APJ). ELA decreases FoxM1 mRNA levels in the heart tissues of transaortic constricted mice [86, 87]. FoxM1 forms a repressor complex with Brg1 and inhibits ACE2 transcription [71]. Treatment of ELA alleviates transaortic constriction surgery-induced heart failure and attenuates Ang II-induced cardiac hypertrophy in mice [86]. Ang II treatment inhibits ACE2 mRNA levels in rat aortic fibroblast; the Ang II-induced ACE2 mRNA levels are restored by treatment with either ELA or FGF21 [88]. The mechanism of ELA or FGF21-enhanced ACE2 mRNA levels in aortic fibroblasts remains unclear.

##### Interleukin 1β (IL-1β)

Interleukin 1β (IL-1β) is a macrophage-secreted proinflammatory cytokine that belongs to the interleukin 1 (IL-1) family. IL-1 induction is positively correlated with cartilage degradation [89]. ACE2 cleaves Ang II into angiotensin 1–7 (Ang-(1–7)), which improves glucose metabolism [90]. ACE2 overexpression in human A549 lung adenocarcinoma cells attenuates metastasis to the lungs and the liver of recipient mice [91]. IL-1β treatment increases mRNA levels of ACE2 and MAS (the Ang-(1–7) receptor) in human U-2 OS and MNNG-HOS osteosarcoma cells [92]. Moreover, IL-1β treatment inhibits the proliferation and migration of U-2 OS cells and MNNG-HOS cells [92]. Taken together, IL-1β may inhibit osteosarcoma migration through enhancement of ACE2 levels.

##### Chitinase 3-like-1 (CHI3L1)

Chitinase 3-like-1 (CHI3L1), a member of the glycosyl hydrolase 18 family, is secreted by activated macrophages, neutrophils, chondrocytes, and synovial cells. CHI3L1 is highly expressed in the lung tissues of patients with aging, cardiovascular disease, and chronic lung disease [93–100]. Moreover, circulating CHI3L1 levels are induced in aging, hypertension, and severe COVID-19 [10]. CHI3L1 transgene induces ACE protein levels in the lung tissues of CH3IL1 transgenic mice; CHI3L1 recombinant protein stimulates ACE2 mRNA levels in human Calu3 lung epithelial cells [10]. The CH3IL1-induced ACE2 facilitates infection of SARS-CoV-2 pseudovirus into Calu-3 lung epithelial cells [10]. Conversely, inhibition of CH3IL1 by monoclonal antibody or the small-molecule inhibitor kasugamycin blocks CH3IL1-induced ACE2 mRNA levels in vitro and in vivo [10]. These findings suggest that CH3IL1 monoclonal antibody and small-molecule inhibitors may be potential therapeutics for ACE2-mediated diseases.

#### Other inhibitors of ACE2 mRNA levels

##### Nuclear factor erythroid 2-related factor 2 (Nrf2)

Nuclear factor erythroid 2-related factor 2 (Nrf2) is a transcription factor that belongs to the basic leucine-zipper protein family. ACE2 mRNA and protein levels are decreased in renal proximal tubular cells (RPTCs) of aged type 1 diabetes mice [24]. Treatment of high-concentration glucose increases Nrf2 protein levels in rat RPTCs, whereas ACE2 transcription is reduced in the high-glucose-treated RPTCs [24]. The high-glucose-reduced ACE2 protein activity and ACE2 mRNA levels in rat RPTCs are restored by either Nrf2 siRNA knockdown or Nrf2 inhibitor [24]. The decreased ACE2 mRNA and protein levels in RPTCs of type 1 diabetes mice are reversed by Nrf2 knockout; nephropathy in the symptomatic mice is also attenuated by Nrf2 knockout [24]. These findings suggest that the enhancement of ACE2 by Nrf2 inhibitor may be a therapeutic strategy for diabetic nephropathy.

##### miR-143

miR-143 levels are increased in the aortas of spontaneously hypertensive rats (SHRs), whereas ACE2 mRNA and protein levels are decreased [101]. ACE2 activity and protein levels are elevated by exercise training in the heart of normotensive rats [102]. Moreover, ACE2 mRNA and protein levels are increased in the aortas of SHRs after exercise training [101]. In contrast, miR-143 levels are decreased by exercise training in the heart of normotensive rats and in the aorta of SHRs [101, 102]. Exercise training mitigates aortic remodeling in SHRs [101]. These findings suggest that the induction ACE2 levels by exercise training could be a non-pharmacological regimen to prevent hypertension.

##### Ang II type 1 receptor (AT1R)

ACE2 mRNA and protein levels are decreased in the heart of hypertensive cardiopathy patients and the kidney of hypertensive nephropathy patients [103]. Ang II type 1 receptor (AT1R) mediates the vasoconstriction in the rennin-angiotensin system (RAS). Ang II decreases ACE2 mRNA and protein levels in the human HK2 kidney tubular epithelial cells [103]. Inhibition of AT1R by the antagonist losartan rescues the Ang II-mediated ACE2 reduction in human HK2 cells [103]. Moreover, the Ang II-decreased ACE2 levels are recovered by the p38 inhibitor SB203580 and the ERK1/2 inhibitor PD98059 [103]. These findings show that the Ang II-AT1R axis decreases ACE2 levels through AT1R in human kidney epithelial cells.

##### Interleukin 13 (IL-13)

ACE2 expression is downregulated by the Th2 cytokine interleukin 13 (IL-13) [64, 66, 104]. IL-13 is induced in allergic diseases such as allergic rhinitis and type 2 asthma [105]. The weighted gene co-expression network analysis shows that ACE2 gene expression is inversely correlated with allergic asthma in children [64]. Single cell RNA sequencing (scRNA-seq) analysis shows that ACE2 mRNA levels are decreased in the IL-13-stimulated tracheal airway basal epithelial cells and intermediate secretory cells of asthma patients [64]. IL-13 stimulation decreases ACE2 mRNA and protein levels in human primary airway epithelial cells derived from asthma patients [64, 104]. These results indicate that ACE2 gene expression is reduced by IL-13 signaling in human airway epithelial cells [64].

##### Transforming growth factor-β (TGF-β)

Hyperglycaemia-induced overexpression of transforming growth factor-β (TGF-β) overproduction in the renal tissues contributes to the pathogenesis of diabetic nephropathy [106]. Diabetic nephropathy patients show a decrease of ACE2 mRNA/protein levels in the glomerular and renal tubular cells [107]. ACE2 mRNA and protein levels are decreased by TGF-β treatment in rat NRK-52E renal proximal tubular cells [108]. Pre-treatment of the TGF-β receptor inhibitor SB431542 restores the high-glucose-inhibited ACE2 mRNA levels in rat NRK-52E cells [108]. These findings show that TGF-β signaling inhibits ACE2 gene expression in renal tubular cells.

##### Estrogen

Statistics analysis shows that the intensive care rate and death rate for female COVID-19 patients are lower than those of male COVID-19 patients [109, 110]. Estrogen treatment decreases ACE2 mRNA levels in normal human bronchial epithelial cells [12]. ACE2 mRNA levels, protein levels, and enzyme activity are higher in the kidneys of male mice than those of female mice [11]. In addition, the estrogen 17β-estradiol (E2) reduces renal ACE2 enzyme activity in the sex-chromosome-independent manner in gonadectomized mice [11]. These findings show that ACE2 levels and activity in lungs or kidneys are inhibited by estrogen. In contrast, estrogen treatment increases ACE2 mRNA and protein levels in the human atrial tissues [111]. Thus, the effects of estrogen on ACE2 levels may be tissue-specific.

##### Myc-interacting zinc finger protein-1 (Miz1)

Myc-interacting zinc finger protein-1 (Miz1, also named ZBTB17) protein levels are decreased in the lung epithelial cells of patients with severe chronic obstructive pulmonary disease. Miz1 is a transcriptional repressor that inhibits IL-6 and IL-1β transcription [112, 113]. Moreover, ACE2 mRNA levels are increased in MIZ1-deficient mice [113]. IL-6/STAT3 signaling upregulates ACE2 transcription [37, 38]. IL-1β signaling leads to the enhancement of ACE2 levels [66, 92]. These findings suggest that Miz1 may decrease ACE2 levels by inhibiting IL-6 and IL-1β signaling in lung epithelial cells.

##### Hypoxia-inducible factor-1α (HIF-1α)

Hypoxia-inducible factor-1α (HIF-1α) is a transcription factor that is induced under hypoxia [114, 115]. Chronic hypoxia contributes to hypoxic pulmonary hypertension [114]. ACE2 downregulation is associated with human pulmonary arterial pressure [116]. Overexpression of HIF-1α decreases ACE2 protein levels in human pulmonary artery smooth muscle cells (hPAMCs) under normoxia [114]. The hypoxia-stimulated ACE2 protein levels in hPASMCs are further enhanced by HIF-1α siRNA knockdown [114]. These findings indicate that ACE2 gene expression is inhibited by HIF-1α [114].

##### Tumor necrosis factor-α (TNF-α)

Meta analysis shows that ACE2 mRNA levels are decreased in the small bowel of Crohn’s disease (CD) patients and increased in the colon tissues of ulcerative colitis (UC) patients [117]. ACE2 mRNA levels are restored in the ileum of CD patients by anti-tumor necrosis factor-α (TNF-α) antibody therapy [117]. In contrast, ACE2 mRNA levels are decreased in the colon of UC patients after anti-TNF-α antibody treatment [117]. These results suggest that ACE2 mRNA levels may be decreased by TNF-α signaling in the ileum of CD parents, but increased by TNF-α signaling in the colon of UC patients.

### Transcriptional regulation of ACE2 mRNA isoforms

RNA sequencing database analysis shows that the truncated ACE2 isoform is detected in human primary nasal epithelial cells, bladder cancer cells, and Sendai virus-infected human cancer cells [118, 119]. The truncated ACE2 isoform is initiated from the novel first exon in intron 9 of the full-length ACE2 transcript [118, 119]. This truncated ACE2 transcript encodes a 459 a.a. ACE2 protein (residues #347 to 805) with a molecular weight of 52 kDa [118, 119]. Analysis of UCSC Genome Browser database shows that the truncated ACE2 isoform only exits in primates among 100 vertebrate species [118]. The mRNA levels of the truncated ACE2 are lower than full-length ACE2 in multiple human organs; in contrast, the mRNA levels of the truncated ACE2 are higher than full-length ACE2 only in human kidneys [119]. IFN-β or IFN-γ signaling stimulates the promoter activity of the truncated ACE2 transcription, but not the full-length ACE2 transcription in human HepG2 hepatoma cells [118]. The promoter region of the truncated ACE2 isoform contains two STAT-binding elements (− 44 to − 34 bp and − 33 to − 22 bp) that are responsible for IFN-β or IFN-γ-stimulated transcription [118]. Similarly, mRNA levels of the truncated ACE2 isoform are increased by the treatment of IFN-α or IFN-λ3 in human primary bronchial epithelial cells; the mRNA levels are also increased by IFN-β or IFN-λ1-3 in human colon or ileum organoid [118]. Moreover, the truncated ACE2 mRNA levels in human primary airway epithelial cells are induced by infection of rhinovirus or H3N2 influenza virus, which also induces gene expression of IFNs and IFN-stimulated genes [118]. Interestingly, SARS-CoV-2 infection enhances the mRNA levels of the truncated ACE2 isoform in human colon cancer cell lines or primary bronchial epithelial cells [118, 119]. Notably, the truncated ACE2 protein loses both its SARS-CoV-2 spike (S) protein-binding activity and carboxypeptidase activity [118]. Collectively, the truncated ACE2 isoform may not regulate the cell entry of SARS-CoV-2. The role of SARS-CoV-2-induced the truncated ACE2 isoform in the COVID-19 pathogenesis needs to be further investigated.

## Post-translational modification and regulation of ACE2 protein in chronic disease

### Phosphorylation

#### AMP-activated protein kinase (AMPK)

**Fig. 4:**
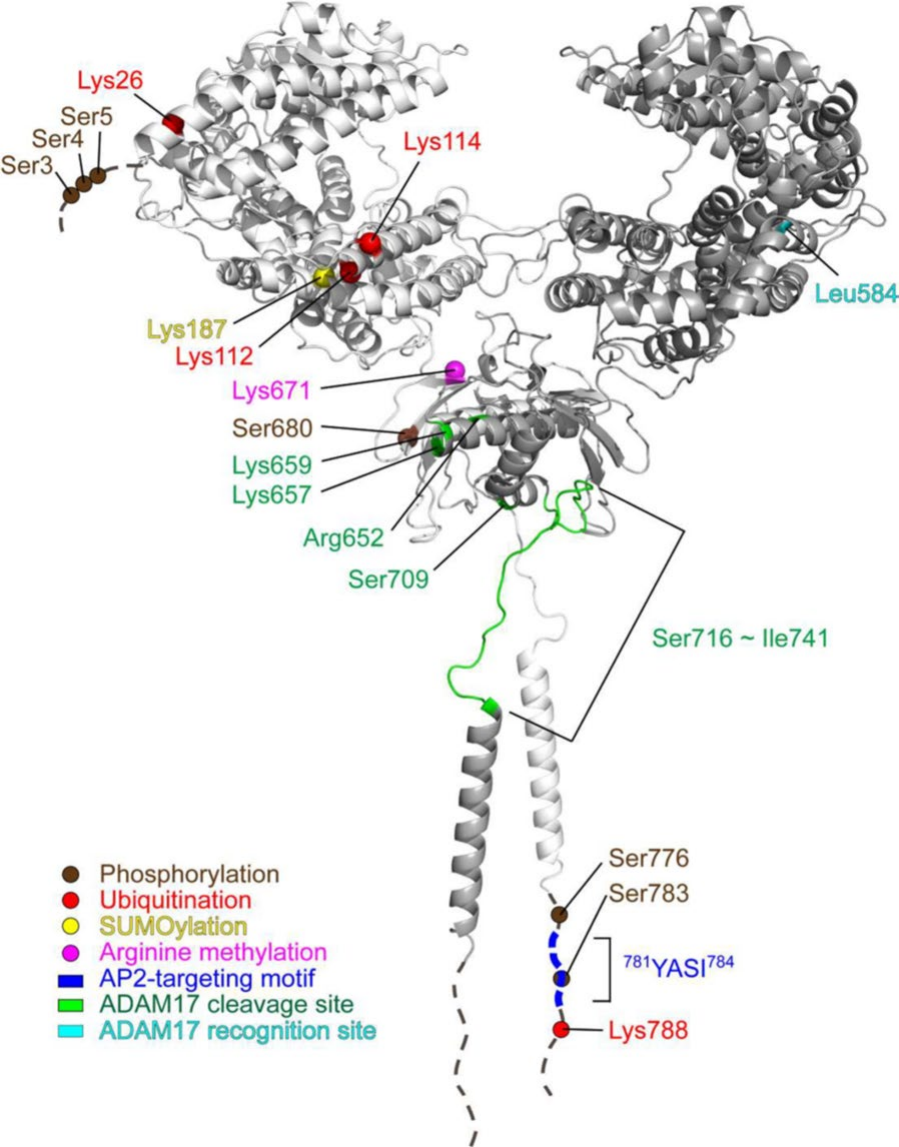
A three-dimensional model depicts ACE2 homodimer containing post-translational modification sites. Cα atoms of modified ACE2 residues are marked by spheres. ^781^YASID^784^ is the AP2-targeting motif for lysosomal degradation. ACE2 Arg652, Lys657, Lys659 residues, and Ser716 to Ile741 region are the reported ADAM17 cleavage sites. The ACE2 protein structure is obtained from Protein Data Bank (ID: 6M17) and operated by homology modeling using SWISS-MODEL. Dash lines represent undetermined structure of ACE2 protein

Endothelial dysfunction and pulmonary arterial pressure (PAP) contribute to pulmonary hypertension, which is associated with heart failure [116]. The ACE2-mediated renin-angiotensin system (RAS) in endothelial cells controls vasodilation, antifibrosis, and antihypertrophy [120], leading to the protection against pulmonary hypertension [116]. ACE2 levels are inversely correlated with human PAP levels [116]. Administration of recombinant ACE2 mitigates symptoms of pulmonary hypertension mice [116], suggesting that ACE2 induction is a therapeutic approach for human pulmonary hypertension. The ACE2 protein is phosphorylated by the serine/threonine kinase AMPK, resulting in the enhancement of ACE2 protein levels [116]. Human ACE2 Ser680 is a highly conserved residue in human, mouse, rat, and rabbit ACE2 proteins. AMPK phosphorylates human ACE2 at Ser680 residue (Fig. 4), leading to inhibition of ACE2 Lys48-linked ubiquitination and prevention of ACE2 proteasomal degradation [116]. ACE2 protein levels are increased by ACE2 (S680D) phosphomimetic mutation, but decreased by ACE2 (S680A) phospho-deficient mutation [116]. Moreover, ACE2 (S680D) phosphomimetic mutation-knockin mice display high ACE2 protein levels in lung tissues; the knockin mice are resistant to the induction of pulmonary hypertension [116]. Consistently, ACE2 Ser680 phosphorylation and AMPK activation are reduced in the lung tissues of patients with idiopathic pulmonary arterial hypertension (IPAH) patients [116]. In addition, AMPK levels are induced by overexpression of the deacetylase SIRT6 [121]. SIRT6 overexpression results in AMPK activation and ACE2 induction in myocardial cells of hypertensive rats [122]. These results suggest that AMPK-mediated ACE2 phosphorylation and subsequent ACE2 protein stabilization prevent the lung or heart damage in pulmonary hypertension and heart failure.

### Ubiquitination

#### Mouse double minute 2 homolog (MDM2)

The E3 ligase, mouse double minute 2 homolog (MDM2, also known as HDM2) is an oncogene that induces ubiquitination and degradation of the p53 tumor suppressor protein [123, 124]. MDM2 mRNA and protein levels are increased in the lung tissues and artery endothelial cells from pulmonary arterial hypertension (PAH) patients [125]. In contrast, ACE2 protein levels are decreased in the lung tissue and artery endothelial cells from PAH patients [125]. MDM2 induces ACE2 ubiquitination at Lys788 residue (Fig. 4), leading to the proteasomal degradation of ACE2 protein [125]. Inhibition of MDM2 increases ACE2 levels in the lung tissues of mice and alleviates pulmonary hypertension of mice [125]. Interestingly, ACE2 ubiquitination levels are enhanced by ACE2 (S680L) phospho-deficient mutation and decreased by ACE2 (S680D) phosphomimetic mutation [125]. ACE2 Ser680 could be phosphorylated by the kinase AMPK [116]. Collectively, AMPK-induced ACE2 Ser680 phosphorylation may block MDM2-mediated ACE2 ubiquitination and degradation. Adenovirus-transduced ACE2 (K788R) ubiquitination-deficient mutant attenuates hypoxia-induced pulmonary hypertension in AMPK knockout mice [125]. These findings suggest that MDM2 inhibitors or AMPK agonists may be therapeutics for pulmonary hypertension.

#### Neural precursor cell expressed developmentally down-regulated 4-like (NEDD4L)

Bioinformatics analysis shows that the top two putative E3 ubiquitin ligases for ACE2 protein are MDM2 and neural precursor cell expressed developmentally down-regulated 4-like (NEDD4L) [126]. Overexpression of Ang II and AT1R enhances ACE2 ubiquitination in human HEK293T cells [126]. Enhancement of ubiquitination by the deubiquitinase inhibitor PR619 decreases ACE2 protein levels in HEK293T cells [126]. NEDD4L and ACE2 protein levels are inversely correlated in the brain, the heart, and kidneys of Ang II-treated male mice [126]. Ang II-decreased ACE2 levels are restored by NEDD4L-deficient mutation or NEDD4L siRNA knockdown in human HEK293T cells and human aorta endothelial cells [126]. Downregulation of ACE2 through Ang II is slightly recovered by ACE2 (K769/770/771/773/788R) ubiquitination-deficient mutation in HEK293T cells [126]. Mean arterial pressure of Ang II-infused mice is mitigated by adenovirus-transduced ACE2 (K769/770/771/773/788R) ubiquitination-deficient mutation [126]. These findings show that Ang II may decrease ACE2 protein levels through NEDD4L-mediated ubiquitination, contributing to hypertension in male.

#### S-phase kinase-associated protein-2 (Skp2)

ACE2 protein levels are higher in human non-smokers than those of smokers [127]. Among the confirmed COVID-19 patients, smoker COVID-19 patient subpopulation is relatively small [128, 129]. Cigarette smoke extract (CSE) and carcinogen benzo(a)pyrene (BaP) drastically inhibit ACE2 protein levels in lung epithelial cells by inducing proteasomal and lysosomal degradation of ACE2 protein [127]. Notably, CSE and BaP slightly enhance ACE2 mRNA levels [127]. Moreover, BaP stimulates AhR-mediated S-phase kinase-associated protein-2 (Skp2) gene expression in lung epithelial cells [127]. Skp2 is an E3 ubiquitin ligase that regulates cell cycle by inducing degradation of tumor suppressor genes such as p21, p27, and p57 [130]. Skp2 induces ACE2 ubiquitination and degradation [127]. Treatment of the CDK4/6 inhibitor palbociclib decreases Skp2 mRNA and protein levels, leading to the upregulation of ACE2 protein levels [131]. Tobacco smoke also enhances Skp2 protein levels and decreases ACE2 protein levels in the lung tissues of mice [127]. The BaP-induced ACE2 degradation in lung epithelial cells is reversed by Skp2 siRNA knockdown [127]. Consistently, Skp2 protein levels are inversely correlated with ACE2 protein levels in the lung tissues of human lung cancer patients [127]. Inhibition of Skp2 by palbociclib enhances SARS-CoV-2 pseudovirus infection in human Huh7 hepatoma cells and monkey Vero E6 kidney cells [131]. In addition, CSE or BaP-induced ACE2 downregulation suppresses SARS-CoV-2 pseudovirus entry into lung epithelial cells [127], suggesting that infection efficiency of SARS-CoV-2 is decreased in smokers. Importantly, the disease severity of smoker COVID-19 patients is increased compared to those of non-smokers [132]; therefore, tobacco use may exacerbate COVID-19 severity.

#### Ubiquitin-specific proteases 4 (USP4)

Ubiquitin-specific proteases 4 (USP4) is a deubiquitinating enzyme [133]. Wang et al. searched the GEPIA database and reported that USP4 mRNA levels are significantly decreased in lung adenocarcinoma patients [134]; USP4 downregulation is correlated with poor survival of lung cancer [134]. In addition, the authors searched the GEPIA database and concluded that ACE2 levels are positively correlated with the stage of human lung cancer [134]. The authors proposed that USP4 overexpression enhances ACE2 protein levels [134]; nevertheless, the supporting data are lacking.

### Ectodomain shedding

The soluble ACE2 protein (around 70 to 105 kDa) missing the C-terminal tail of ACE2 retains the carboxypeptidase activity [135–137]. A disintegrin and metalloprotease 17 (ADAM17) is a protease that cleaves ACE2 protein and mediates shedding of ACE2 ectodomain as soluble ACE2 (sACE2) [135–137]. Conversely, inhibition of ADAM17 by inhibitors or siRNA knockdown reduces sACE2 shedding [135, 136]. Three publications reported that the ADAM17 cleavage sites on the ACE2 protein are at the region between ACE2 Ser716 and Ile741 residues [136] or individual Ser709 [138], Arg652, Lys657, and Lys659 [139] residues (Fig. 4). Moreover, ACE2 (L584A) mutation inhibits sACE2 in human HEK293 cells, indicating that Leu584 residue may be part of recognition motif for ADAM17 [136] (Fig. 4).

sACE2 protein is detected in the bronchoalveolar lavage fluid from human healthy volunteers, as well as in the supernatants of human primary airway epithelial cells [136]. The soluble ACE2 protein levels are increased in the urine from human type 1 diabetes (T1D) and type 2 diabetes (T2D) patients [140, 141], as well as diabetic mice [19, 20]. Induction of urinary sACE2 is correlated with disease severity of diabetic kidney disease [142], which could be due to the accumulation of sACE2 protein in the kidney [129]. In addition to diabetic nephropathy, sACE2 and ADAM17 protein levels are increased in the peripheral blood of patients with myocardial infarction-induced heart failure and in the culture supernatants of H_2_O_2_-damaged rat cardiomyocytes [143]. The sACE2 levels and cell apoptosis in these rat cardiomyocytes are decreased by ADAM17 siRNA knockdown; the recovered phenotypes may be due to the increase of cellular ACE2 protein in cardiomyocytes by ADAM17 knockdown [143]. Collectively, induction of soluble ACE2 protein contributes to the pathogenesis of diabetic nephropathy and myocardial infarction-induced heart failure.

## Post-translational modification and regulation of ACE2 protein in COVID-19

### Phosphorylation

#### Casein kinase 1α (CK1α)

Inactivation of casein kinase 1α (CK1α) can phosphorylate the E3 ubiquitin ligase SPOP (speckle-type BTB–POZ protein)-binding motif of its substrates (e.g., PDK-1 or Friend leukemia integration 1), leading to the induction of a direct interaction between SPOP and its substrates (e.g., PDK-1 or FLI1), as well as subsequent ubiquitin-mediated degradation of the substrates [144, 145]. ACE2 protein sequences contain a consensus SPOP-binding motif Φ-Π-S–S/T-S/T (Φ, nonpolar; Π, polar) [146], ^1^MSSSS^5^ [147] (Fig. 4). Interestingly, ACE2 protein levels are decreased by SPOP shRNA knockdown in human UMRC kidney cancer cells [147], suggesting that SPOP prevents, but not promotes, ACE2 protein from degradation. ACE2 protein levels are also decreased by CK1α shRNA knockdown or CK1α inhibitor treatment [147]. The SPOP-ACE2 interaction is blocked by a phospho-deficient mutation of the SPOP motif on the ACE2 protein [147]. Moreover, ACE2 ubiquitination is reduced by SPOP overexpression [147]. These results suggest that CK1α phosphorylates ACE2 and induces the binding between SPOP and SPOP motif of ACE2, leading to the prevention of ACE2 protein from other E3 ligase-mediated protein degradation. ACE2 protein is stabilized through CK1α-induced phosphorylation on ACE2 Ser3, Ser4, and Ser5 residues [147] (Fig. 4). Furthermore, infection of SARS-CoV-2 pseudovirus (S protein) to human UMRC kidney epithelial cells is reduced by treatment of the CK1α inhibitor lenalidomide [147]. These findings suggest that lenalidomide treatment may prevent SARS-CoV-2 infection or re-infection by reducing ACE2 phosphorylation and inducing ACE2 degradation [147]. Notably, the ACE2 E3 ligase MDM2 and NEDD4, identified from chronic lung diseases, has been ruled out as the SPOP-competing E3 ligases for ACE2 protein in kidney cells [147].

#### NUAK family kinase 2 (NUAK2)

NUAK family kinase 2 (NUAK2) is an AMPK-related kinase that enhances the formation of actin stress fibers by increasing the conversion of filamentous actin to globular actin [148]. Infection of live SARS-CoV-2 virus or treatment of SARS-CoV-2 spike (S) protein increases NUAK2 mRNA levels in human A549 epithelial cells [149]. NUAK2 is required for the maintenance of the surface ACE2 proteins and the entry of SARS-CoV-2 virus [149]. Interestingly, SARS-CoV-2-infected A549 epithelial cells secrete S proteins or other messenger molecules to increase NUAK2 and maybe ACE2 levels in bystander cells, leading to promoting of viral spread [149]. Notably, NUAK2 induces cell surface ACE2 levels in an ACE2-Ser680-phosphorylation independent manner [149]; it is possible that NUAK2 enhances ACE2 protein levels by phosphorylating ACE2 at other serine/threonine residues such as Ser776 or Ser783, previously identified as MAP4K3/GLK-phosphorylation sites [28]. Collectively, NUAK2 induces ACE2 protein levels on cell surface, resulting in enhancement of SARS-CoV-2 infection [149].

#### MAP4K3 (GLK)

The serine/threonine kinase MAP4K3 (also named GLK [150]) belongs to the mammalian Ste20-like serine/threonine kinase family [151]. The scRNA-seq analyses from two independent COVID-19 patient cohorts show that GLK is induced in macrophages and epithelial cells from airway tissues of COVID-19 patients [28]. GLK interacts with and phosphorylates PKC-θ in T cells, leading to activation of IKKβ/NF-κB and induction of T-cell activation [152]. Overexpression of GLK in T cells causes overproduction of proinflammatory cytokine IL-17A and downregulation of Treg differentiation, contributing to autoimmune diseases [152–154]. In epithelial cells, GLK overexpression is highly correlated with the recurrence of hepatoma and lung cancer [155–157].

**Fig. 5:**
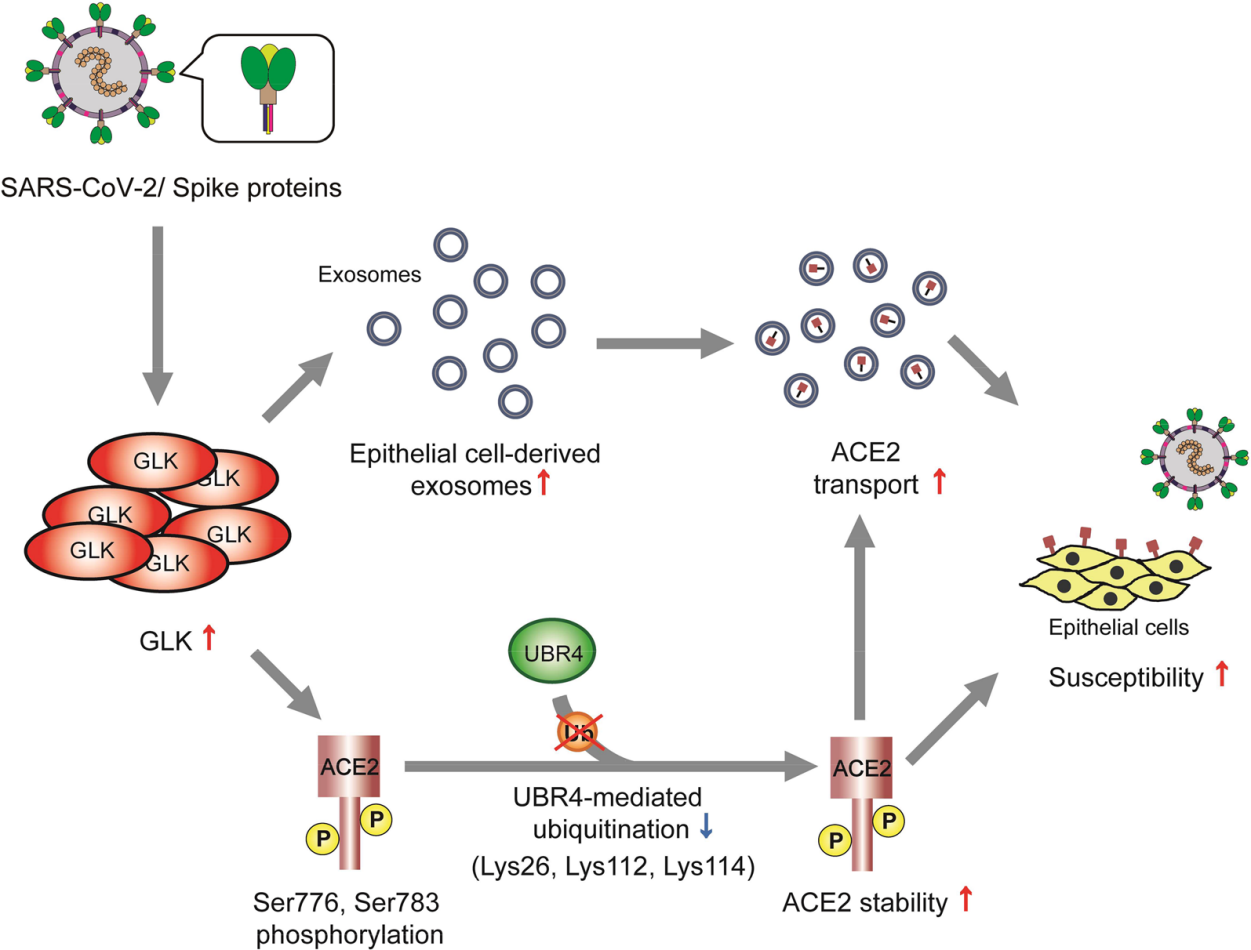
Upregulation of ACE2 by SARS-CoV-2-induced GLK (MAP4K3) in epithelial cells. Spike protein of SARS-CoV-2 induces GLK (MAP4K3) mRNA levels in lung epithelial cells. GLK enhances ACE2 stability by phosphorylating ACE2 at Ser776 and Ser783 residues, leading to dissociation of ACE2 and the E3 ubiquitin ligase UBR4. Moreover, SARS-CoV-2-induced GLK increases epithelial cell-derived exosomes, resulting in an induction of exosomal ACE2. ACE2 proteins are transported by epithelial cell-derived exosomes to other epithelial cells, inducing cell susceptibility to SARS-CoV-2 virus

GLK mRNA levels are induced by SARS-CoV-2 spike (S) proteins in human HCC827 lung epithelial cells [28]. As an ACE2-interacting protein, the kinase GLK stabilizes ACE2 protein by inducing ACE2 phosphorylation and inhibiting ACE2 Lys48-linked ubiquitination [28]. The induction of ACE2 protein also occurs in the lung tissues of mice infected with either live SARS-CoV-2 virus or SARS-CoV-2 pseudovirus [28]; conversely, ACE2 protein levels in murine lung tissues are decreased by the GLK inhibitor verteporfin or GLK ablation [28]. Consistently, ACE2 protein concentration in the plasma is induced over time stratified by the severity of human COVID-19 patients [27]. Similarly, ACE2 protein levels are increased in the serum exosomes of COVID-19 patients, while ACE2 protein levels are decreased to normal levels in recovered patients with COVID-19 [28]. Mechanistically, GLK phosphorylates ACE2 at Ser776 and Ser783 residues [28] (Fig. 4), leading to the dissociation of ACE2 and the E3 ubiquitin ligase UBR4 [28]. Consistently, GLK-induced in ACE2 Ser776/Ser783 phosphorylation is detectable in the serum exosomes from human COVID-19 patients, whereas AMPK-induced ACE2 Ser680 phosphorylation is not detectable [28]. Collectively, SARS-CoV-2-induced GLK phosphorylates and stabilizes ACE2 protein in epithelial cells, contributing to severe COVID-19 (Fig. 5). Notably, besides GLK, SARS-CoV-2 spike proteins also induce the levels of the kinase NUAK2, leading to the induction of cell surface ACE2 levels and subsequent enhancement of SARS-CoV-2 infection [149].

### Exosomal ACE2

Extracellular vesicles are reported to participate in several physiological and pathological processes [158]. Exosome is one of the extracellular vesicle subtypes with the size around 30 to 150 nm in diameter [159]. A model was proposed to describe the mechanism of extracellular vesicles uptake [160]. First, the extracellular vesicle-carried protein binds to the proteoglycan on the cell surface. Next, the interacting protein of the extracellular vesicle-carried protein on cell surface binds to the proteoglycan, promoting the extracellular vesicle uptake [160, 161]. Several viruses take advantage of the exosome for transporting viral and cellular elements that are beneficial for viral infections [158]. Interestingly, the vesicle trafficking pathway-related genes are closely associated with SARS-CoV-2 infection through the gene ontology enrichment analysis [162]. The RNAi screening analysis shows a reduction of SARS-CoV-2 infection by knockdown of vesicle trafficking pathway-related genes in human HK-2 kidney tubular epithelial cells [162]. The ACE2 expression is low abundant in human airway epithelial cells under normal physiological condition [9, 25]. COVID-19 patients display the induction of ACE2-containing exosomes in their sera [28]. Exosome-transported ACE2 protein can increase ACE2 levels in the exosome-recipient epithelial cells, facilitating the enhancement of SARS-CoV-2 infection in vitro and in vivo [28].

Single-cell RNA sequencing (scRNA-seq) analysis shows that MAP4K3 (GLK) mRNA levels are induced in lung epithelial cells of severe COVID-19 patients [28]. The transcripts of genes that are related to vesicle, intracellular vesicle, and extracellular vesicle are upregulated in GLK-overexpressing epithelial cells [28]. GLK expression is induced by stimulation of SARS-CoV-2 spike (S) protein in epithelial cells or infection of SARS-CoV-2 pseudovirus in mice [28]. The epithelial cell-derived exosome particle numbers are highly increased by GLK overexpression in human HCC827 lung epithelial cells [28]. Moreover, ACE2 protein levels are increased in the serum exosomes from COVID-19 patients [28]. Notably, exosomal ACE2 from COVID-19 patients contains the full-length protein sequence, including ectodomain, transmembrane domain, and C terminal cytoplasmic tail [28], but not soluble form of ACE2 (sACE2). The GLK-induced ACE2-containing exosomes enhance the susceptibility of recipient lung epithelial cells and recipient mice to infection of SARS-CoV-2 pseudovirus [28]. These results show that the GLK-induced exosomal ACE2 is a functional receptor for SARS-CoV-2 S protein, leading to enhancement of viral infection [28] (Fig. 5).

A conflicting result shows that ACE2-containing exosomes could be used as a decoy to attenuate SARS-CoV-2 infection in cultured cells [163]. However, only simultaneous co-treatment of the decoy (ACE2-containing exosomes) with SARS-CoV-2 pseudovirus could achieve neutralizing effects in vitro [163]. Furthermore, the in vitro result has not been validated using animal models. Thus, it is unclear regarding the feasibility of using ACE2-containing exosomes as a decoy to prevent SARS-CoV-2 infection.

### Ubiquitination

#### Ubiquitin ligase E3 component N-recognin 4 (UBR4)

The ubiquitin ligase E3 component N-recognin 4 (UBR4) is identified as an ACE2-interacting protein [28]. UBR4 induces Lys48-linked ubiquitination of ACE2 protein at Lys26, Lys112, and Lys114 residues [28] (Fig. 4). Conversely, UBR4-induced Lys48-linked ubiquitination of ACE2 is decreased by ACE2 (K26/112/114R) ubiquitination-deficient mutation, while UBR4-inhibited ACE2 protein levels are restored by ACE2 ubiquitination-deficient mutation. Interestingly, the interaction between ACE2 and UBR4 is blocked by the kinase GLK (MAP4K3) [28]; GLK is overexpressed in airway epithelial cells from human COVID-19 patients and in SARS-CoV-2 spike (S) protein-stimulated human HCC827 lung epithelial cells [28]. The GLK-induced ACE2 phosphorylation reduces Lys48-linked ubiquitination of ACE2 protein, resulting in the induction of ACE2 protein levels. Consistently, ACE2 protein levels are induced in the lung tissues of mice infected with either SARS-CoV-2 pseudovirus or live SARS-CoV-2 virus [28]. The SARS-CoV-2 pseudovirus-induced ACE2 protein levels in the lung tissues of mice are decreased by the GLK inhibitor verteporfin [28]. Collectively, SARS-CoV-2-induced GLK phosphorylates and subsequently stabilizes ACE2 protein by blocking UBR4-ACE2 interaction and reducing UBR4-mediated ACE2 Lys48-linked ubiquitination, leading to the enhancement of SARS-CoV-2 infection [28] (Fig. 5).

#### Ubiquitin carboxyl-terminal hydrolase L1 (UCHL1)

Ubiquitin carboxyl-terminal hydrolase L1 (UCHL1) is a deubiquitinase that removes ubiquitins from its target proteins [164]. SARS-CoV-2 pseudovirus (S protein) increases ACE2 protein levels in human Calu-3 lung epithelial cells and human Bease2B bronchial epithelial cells [29]. Results from deubiquitinase library screening show that SARS-CoV-2 pseudovirus-induced ACE2 overexpression is reduced by UCHL1 siRNA knockdown in human Bease2B epithelial cells [29]. Ubiquitination of ACE2 protein is enhanced by UCHL1 siRNA knockdown in human Bease2B epithelial cells [29]. In contrast, ACE2 protein levels are increased by UCHL1 overexpression in human Bease2B epithelial cells [29]. Inhibition of UCHL1 by its inhibitor LDN-57444 inhibits SARS-CoV-2 live virus infection of human Calu-3 epithelial cells and human bronchial epithelial cells [29]. These findings indicate that SARS-CoV-2 spike (S) protein stabilizes ACE2 protein through UCHL1-mediated deubiquitination, contributing to enhancement of SARS-CoV-2 infection. These findings also suggest that UCHL1 inhibitors may be potential therapeutics for SARS-CoV-2 infection by downregulating ACE2 proteins.

#### Ubiquitin‐specific peptidase 50 (USP50)

Ubiquitin‐specific peptidase 50 (USP50) is a deubiquitinase that removes ubiquitins from its target proteins [152]. Overexpression of USP50 reduces ACE2 Lys48-linked ubiquitination and enhances ACE2 protein stability in human HEK293T cells [153]. Treatment of vitamin C (Vit C) induces ACE2 Lys48-linked ubiquitination at Lys788 residue, leading to ACE2 degradation in human 2fTGH fibrosarcoma cells and HEK293T cells [153]. The Vit C-induced ACE2 protein degradation is not further enhanced by USP50 knockout, suggesting that Vit C blocks USP50-mediated ACE2 deubiquitination [153]. Treatment of Vit C decreases ACE2 protein levels and inhibits SARS-CoV-2 pseudovirus infection in cultured human Caco-2 colon epithelial cells and humanized ACE2 mouse model [153]. These findings indicate that Vit C inhibits USP50-mediated ACE2 deubiquitination, resulting in downregulation of ACE2 proteins and attenuation of SARS-CoV-2 infection.

### Lysosomal degradation

Clathrin-mediated endocytosis is one of the major endocytosis pathways in mammalian cells [165]. Adaptor protein complex 2 (AP2) binds to the endocytic sorting motifs of the target proteins and triggers clatherin-mediated endocytosis [165]. ACE2 protein levels are decreased in the lung tissues of SARS-CoV-2-infected Syrian hamsters, as well as SARS-CoV-2 spike (S) protein (25 μg)-treated human HEK293A cells [166]. SARS-CoV-2 S protein induces ACE2 protein localization into the endosomes and lysosomes of human HEK293A cells [166]. The SARS-CoV-2 S protein-reduced ACE2 protein levels are restored by treatment of the lysosome inhibitor bafilomycin A1 in human HEK293A cells, suggesting that S protein induces ACE2 lysosomal degradation [166]. The ACE2 cytoplasmic domain contains the AP2-targeting motif (^781^YASI^784^) [166] (Fig. 4). The high-dose S protein-induced ACE2 lysosomal degradation in human HEK293A cells is blocked by AP2-motif-deficient mutation of ACE2 [166]. These results show that high concentration of SARS-CoV-2 S protein induces ACE2 lysosomal degradation through AP2/clatherin-mediated endocytosis in hamster lung tissues and human HEK293A cells.

Notably, this result is inconsistent with two aforementioned publications [28, 29]. ACE2 protein levels are increased by treatment of SARS-CoV-2 pseudovirus (S protein) in human BEAS2B lung epithelial cells [29]. Moreover, ACE2 protein levels in the lung tissues are enhanced by infection of SARS-CoV-2 pseudovirus or SARS-CoV-2 live virus using ACE2-humanized mouse models [28]. Interestingly, stimulation of either SARS-CoV-2 pseudovirus or S protein (1–2 μg) induces mRNA levels of GLK (MAP4K3), which stabilizes ACE2 proteins in human HCC827 lung epithelial cells [28]. These inconsistent results (decreased vs. increased ACE2 levels) may be due to experimental systems used including differences in doses (25 μg vs. 1 μg), stimuli (recombinant S protein vs. SARS-Co-V2 live virus), and animal models (hamsters vs. humanized-ACE2 mice).

### SUMOylation

#### PIAS4 and SENP3

The ubiquitin-like modifier 1 to 5 (SUMO1-5) proteins are conjugated to the target proteins by the SUMO E3 ligase during the SUMOylation process [167]. Conjugating SUMOs to target proteins requires three types of enzymes, including an E1 activating enzyme, an E2 conjugating enzyme, and an E3 ligase [167, 168]. Monomeric SUMO blocks protein degradation by competing with ubiquitin; in contrast, polymeric SUMO promotes protein degradation through the ubiquitin–proteasome degradation pathway [167]. The E3 ligase, protein inhibitor of activated STAT4 (PIAS4) induces SUMO3 SUMOylation of ACE2 protein, resulting in stabilization of ACE2 protein in human lung epithelial cells [169]. Inhibition of ACE2 SUMOylation by PIAS4 inhibitors or by overexpressing the deSUMOylation enzyme SUMO specific peptidase 3 (SENP3) decreases ACE2 protein levels, contributing to the attenuation of SARS-CoV-2 virus infection in vitro or in vivo [169].

The SUMO3 SUMOylation of ACE2 is abolished by ACE2 (K187R) SUMOylation-deficient mutation in human HEK293A cells, indicating that ACE2 SUMO3 SUMOylation occurs at Lys187 residue [169] (Fig. 4). Unexpectedly, the protein levels of ACE2 (K187R) mutant are not affected; this may be due to limited ACE2 SUMOylation in human HEK293A cells. Interestingly, the ACE2 protein degradation induced by the SUMOylation inhibitor ML-792 is restored by treatment of the autophagy inhibitor 3-MA (3-methyladenine) in human Calu-3 epithelial cells [169]. In contrast, the 3-MA-increased ACE2 levels are not downregulated by the treatment of a SUMOylation inhibitor, ML-792 [169]. The data indicate that PIAS4-induced SUMOylation of ACE2 protein prevents autophagy, leading to ACE2 stabilization in lung epithelial cells. Furthermore, SARS-CoV-2 infection of human Calu-3 epithelial cells induces the dissociation of ACE2 with the E3 ligase PIAS4 and the interaction of ACE2 with the autophagy cargo receptor, toll-interacting protein (TOLLIP), resulting in ACE2 downregulation [169]. In addition, suppression of ACE2 SUMOylation enhances ACE2 Lys48-linked ubiquitination in autophagy-inhibited Calu-3 cells [169]. These findings indicate that the PIAS4-induced ACE2 SUMO3 SUMOylation enhances ACE2 protein stabilization by preventing cell autophagy and maybe also by preventing ACE2 ubiquitination. It would be interesting to study whether ACE2 proteins are destabilized by SARS-CoV-2 infection through decreasing ACE2 SUMOylation in vivo.

### Arginine methylation

#### Protein arginine methyltransferase 5 (PRMT5)

In vitro methylation assays and mass spectrometry analysis show that protein arginine methyltransferase 5 (PRMT5) induces ACE2 protein methylation at Arg671 residue [170] (Fig. 4). The interaction between ACE2 protein and receptor-binding domain of SARS-CoV-2 spike (S) protein is reduced by ACE2 (R671K) methylation-deficient mutation or the PRMT5 inhibitor GSK3326595 [170]. Moreover, SARS-CoV-2 pseudovirus infection to human A549 lung epithelial cells is attenuated by treatment of the PRMT5 inhibitor [170]. These findings indicate that PRMT5-induced ACE2 methylation facilitates SARS-CoV-2 infection.

### Glycosylation

Glycoproteins are proteins that are covalently conjugated with glycan chains. Based on different sugar combinations, conjugating enzymes, and linkages, the structure diversity of glycan chains on glycoproteins is more than 10^12^ [171, 172]. ACE2 glycoprotein is the receptor for SARS-CoV-2. The molecular weight of glycosylated ACE2 is around 120 kDa, while the size of non-glycosylated ACE2 is around 100 kDa [119]. Glycosylations on different residues of ACE2 protein can either facilitate or interfere with SARS-CoV-2 infection [173–179]. Four (Asn53, Asn90, Asn322, and Asn546) of the seven N-glycosylation sites within ACE2 extracellular domain regulate the interaction of ACE2 with SARS-CoV-2 spike (S) protein [173–179]. These four residues of ACE2 protein are adjacent to the binding interface between ACE2 and spike (S) protein. Bioinformatics, molecular dynamics (MD) simulation, and modeling data show that glycan chains of Asn53, Asn90, Asn322, and Asn546 residues on ACE2 protein contact the glycan chains of multiple residues on spike (S) protein of SARS-CoV-2 [173–175]. MD simulations of glycosylated ACE2 protein show that ACE2 Asn90 N-glycosylation interferes with the binding of S protein to ACE2 protein [173, 174, 176–179]. Interestingly, mutations of ACE2 Asn90 and Thr92 residues affect Asn90 N-glycosylation due to the disruption of the consensus N-glycosylation motif (NXS/T, X denotes any amino acid) [179]. For example, both ACE2 N90Q and T92Q mutations enhance the binding affinity of ACE2 to S protein [179]. Notable, the entry of live SARS-CoV-2 virus into ACE2-overexpressing HEK293 cells is significantly enhanced by ACE2 (N90A or N90S) glycosylation-deficient mutation [176]. Similarly, the infection of live SARS-CoV-2 virus into ACE2-overexpressing Vero E6 cells is enhanced by ACE2 (N322Q) glycosylation-deficient mutation [174]. These findings indicate that ACE2 Asn90/322 N-glycosylation hampers its binding to S protein, resulting in the reduction of SARS-CoV-2 infection.

In contrast, unlike N-glycosylation of ACE2 Asn90 and Asn322, MD simulation data show that ACE2 Asn53 N-glycosylation facilitates the binding of S protein to ACE2 protein [173, 174]. The binding affinity of the ACE2-S interaction is decreased by ACE2 (N53A or N53S) glycosylation-deficient mutation [176]. Furthermore, the entry of live SARS-CoV-2 virus into ACE2-overexpressing HEK293 cells is significantly reduced by ACE2 (N53A or N53S) glycosylation-deficient mutation [176]. These findings indicate that ACE2 Asn53 N-glycosylation enhances its binding affinity for S protein, contributing to the induction of SARS-CoV-2 infection. Taken together, N-glycosylation of ACE2 protein either facilitates (Asn53) or interferes (Asn90 and Asn322) with SARS-CoV-2 virus infection.

### Ectodomain shedding

ACE2 ectodomain shedding is mediated by ADAM17 [135–137]. Soluble form ACE2 (sACE2) binds to spike (S) protein of SARS-CoV-2, facilitating cell entry of SARS-CoV-2 through vasopressin receptor-mediated endocytosis [162]. Pre-treatment of recombinant sACE2 enhances the susceptibility of live SARS-CoV-2 virus in multiple human epithelial cell lines [162]. The live SARS-CoV-2 infection is blocked by either inhibition of ACE2 sheddase or ADAM17 siRNA knockdown in human HK-2 epithelial cells [162]. Collectively, soluble ACE2 protein facilitates SARS-CoV-2 infection into host cells. Notably, treatment of pre-mixed SARS-CoV-2 pseudovirus (S protein) with recombinant sACE2 could reduce the cell entry of S protein in human A549 lung epithelial cells [180]. It would be valuable to study the modified soluble ACE2 proteins as potential decoy therapeutics for COVID-19.

## Discussion and conclusion

ACE2 protein is required for maintenance of normal physiological functions, and it is expressed in the vascular endothelial cells, artery smooth muscle cells, cardiomyocytes, renal tubular epithelial cells, and pancreatic islet cells. Reduction of ACE2 in the abovementioned cells contributes to multiple chronic diseases, including hypertension, myocardial infarction, nephropathy, and diabetes [4, 5]. In contrast to the requirement of ACE2 for physiological functions, ACE2 protein is the entry receptor of SARS-CoV and SARS-CoV-2 viruses [6, 7]. Increased ACE2 protein levels are correlated with disease severity of SARS-CoV-2 patients [27, 28]. As ACE2 shows different biological functions in different cells, ACE2 levels are regulated by multiple delicate strategies including transcriptional regulation, post-transcriptional regulation (Tables 2, 3, 4), and post-translational modification (Table 5). ACE2 transcription is positively regulated by several transcription factors including SIRT1 [49], HNFs [33–35], GATA6 [36], STAT1 [65], and STAT3 [37, 38]. K27-acetylated or K4-methylated histone H3 epigenetically induces ACE2 transcription [52, 53]. Other factors such as apelin [85], ELA [88], CHI3L [10], IL-1β [92], IFN-α2, IFN-β, and IFN-γ [8, 65, 66] also increase ACE2 mRNA levels. In contrast, the transcription factor Brg1-FoxM1 complex [71] or the transcription factor ERRα [74] bind to the ACE2 promoter, leading to the reduction of ACE2 transcription. K27-methylated histone H3 also decreases ACE2 levels by binding to the ACE2 gene locus [77]. Furthermore, miR-125b [79], miR-200c-3p [82], miR-421 [81], and miR-483-3p [84] reduce ACE2 mRNA stability by binding to the ACE2 3′UTR. Other factors such as Nrf2 [24], AT1R [103], estrogen [11, 12], Miz1 [113], HIF-1α [114], miR-143 [101, 102], TGF-β [108], and TNF-α [117] also decrease ACE2 mRNA levels. Thus, enhancement of ACE2 levels by either agonists of the identified positive regulators or inhibitors of the identified repressors may help prevention or treatment of chronic diseases.

**Table 2:** Regulation of ACE2 transcription

Regulation of ACE2 transcription		
Name	Description	Ref.
Upregulation		
Ikaros	Ikaros binds to the ACE2 promoter at the − 525 to − 519 bp (5′-ATTTGGA-3′) region, enhancing ACE2 transcription	[30]
HNF1α/1β	HNF1α or HNF1β bind to the − 346 to − 330 (5′-GTATCTTTAACAGCTTT-3′), − 329 to − 312 (5′-CTAGGAAAATATTAACCA-3′), − 259 to − 242 (5′-AGGATTAAAGAATAACGT-3′), and − 921 to − 915 bp (5′-AGTCATA-3′) regions of the ACE2 promoter, inducing ACE2 transcription HNF1α and HNF1β are downregulated in human maturity-onset diabetes of the young (MODY) type 3 and MODY type 5 patients, respectively	[33, 34]
HNF4α	HNF4α binds to the − 7436 to − 7423 (5′-GTGATCTTTGACTC-3′) and − 5533 to − 5520 bp (5′-ATGTACTTTGCTCT-3′) regions of the ACE2 promoter HNF4α and ACE2 mRNA levels are decreased by cyclosporine	[35]
GATA6	GATA6 binds to − 408 to − 403 (5′-TTATCT-3′) and − 351 to − 341 bp (5′-TCCGTGTATCT-3′) regions of the ACE2 promoter, inducing ACE2 transcription GATA6 mRNA levels are increased in the lung samples from COVID-19 patients	[36]
STAT3	STAT3 binds to − 2031 to − 2021 bp (5′-TTCAACCTTTT-3′) region of the ACE2 promoter, enhancing ACE2 transcription Activated STAT3 is positively correlated with the protein levels of ACE2 in lung tissues of pulmonary chronic inflammation or lung cancer patients IL-6/STAT3 signaling stimulates ACE2 transcription in inflammatory diseases ACE2 mRNA levels are inhibited by the IL-6 inhibitor 6-*O*-angeloylplenolin	[37, 38]
SIRT1	SIRT1 binds to − 15,794 to − 15,656, − 15,621 to − 15,521 and − 15,607 to − 15,505 bp regions of the ACE2 promoter, inducing ACE2 transcription	[49]
Histone 3	Acetylated histone 3 binds to the ACE2 promoter, epigenetically enhancing ACE2 transcription	[52]
DYRK1A	DYRK1A increases ACE2 mRNA levels by promoting chromatin accessibility, leading to enhancement of SARS-CoV-2 infection	[62]
Interferon	IFN-α2, IFN-β, and IFN-γ induce ACE2 transcription through STAT1 in virus-infected airway epithelial cells IFN-α, IFN-β, IFN-γ, and IFN-λ1-3 increase truncated ACE2 mRNA isoform IFN levels are increased during viral infection	[8, 65, 66]
SMAD4, EP300, PIAS1, BAMBI	Genome-wide CRISPR gene knockout screening analysis shows that SMAD4, EP300, PIAS1, or BAMBI positively regulates ACE2 mRNA levels	[70]
Downregulation		
Brg1-FoxM1	Brg1-FoxM1 protein complex binds to the ACE2 promoter, leading to inhibition of the ACE2 transcription The upregulation of Brg1 is positively correlated with the disease development of human hypertrophic cardiomyopathy TCA-reduced ACE2 protein levels are recovered by the FoxM1 inhibitor thiostrepton	[71]
ERRα	ERRα binds to the ACE2 promoter, reducing ACE2 transcription	[74]
EZH2	EZH2 induces lysine 27 trimethylation on histon 3 (H3K27me3) and blocks acetylated H3 (H3K27ac) binding to the ACE2 promoter, leading to downregulation of ACE2 gene expression	[77]

The numbering of the human ACE2 promoter is based on Ensembl genome browser (ID: ENST00000252519.8)

**Table 3:** Downregulation of ACE2 mRNA by miRNA

Downregulation of ACE2 mRNA by miRNA		
Name	Description	Ref.
miR-125b	miR-125b binds to the 283 to 289 bp (5′-ucaggga-3′) region in ACE2 3′-UTR, leading to reduction of ACE2 mRNA stability under high glucose concentration The miR-125b expression is elevated in the high glucose-exposed cells High-glucose-decreased ACE2 protein levels are recovered by the anti-miR-125b oligonucleotide	[79]
miR-200c-3p	miR-200c-3p binds to the 165 to 186 bp (5′-auugacauugcuuucaguauuu-3′) region in ACE2 3′-UTR, leading to reduction of ACE2 mRNA stability miR-200c-3p levels are increased in H5N1 influenza virus infected cells H5N1 influenza virus-stimulated miR-200c-3p induction is blocked by NF-κB inhibition H5N1 influenza virus-reduced ACE2 protein levels are increased by the anti-miR-200c-3p oligonucleotides	[82]
miR-421	miR-421 binds to the 300 to 314 bp (5′-guaaaugucuguuga-3′) region in ACE2 3′-UTR, leading to reduction of ACE2 mRNA stability	[81]
miR-483-3p	microRNA library screening shows that miR-483-3p may target and inhibits 3′-UTR of four rennin-angiotensin system (RAS) components, including angiotensinogen, ACE1, ACE2, and the Ang II type 2 receptor	[84]

The numbering of the human ACE2 3′-UTR is based on NCBI Nucleotide database NM_001371415

**Table 4:** Additional regulation of ACE2 mRNA levels

Additional regulation of ACE2 mRNA levels		
Name	Description	Ref.
Upregulation		
Apelin	Active apelin enhances ACE2 transcription in apelin receptor-overexpressing cardiomyocytes Loss of apelin or apelin receptor leads to a reduction of heart contractility in mice	[85]
ELA	Ang II-induced ACE2 mRNA levels are restored by treatment of ELA The ELA expression is low in vascular injury-associated hypertension	[88]
IL-1β	IL-1β treatment increases ACE2 mRNA levels in human osteosarcoma cells	[92]
CH3IL1	CH3IL1 recombinant protein stimulates ACE2 mRNA levels in human lung epithelial cells CH3IL1 is highly expressed in the lung tissues of patients with aging, cardiovascular disease, and chronic lung disease Circulating CH3IL1 levels are increased in aging, hypertension, and severe COVID-19 CH3IL1-induced ACE2 mRNA levels are blocked by the CH3IL1 inhibitor kasugamycin	[10]
Downregulation		
Nrf2	Nrf2 knockout restores ACE2 mRNA and protein levels in renal proximal tubular cells of type 1 diabetes mice Nrf2 levels are increased in the renal proximal tubules of 20-week-old type 1 diabetes mice High-glucose-reduced ACE2 mRNA levels are restored by the Nrf2 inhibitor trigonelline	[24]
miR-143	miR-143 levels are increased in the aortas of spontaneously hypertensive rats, whereas ACE2 mRNA and protein levels are decreased	[101, 102]
AT1R	Ang II-AT1R axis downregulates ACE2 through AT1R in human kidney epithelial cells Ang II-mediated ACE2 mRNA reduction is rescued by the AT1R inhibitor losartan	[103]
IL-13	IL-13 signaling reduces ACE2 gene expression in human airway epithelial cells	[64, 66, 104]
TGF-β	TGF-β signaling inhibits ACE2 gene expression in renal tubular cells TGF-β overproduction in the renal tissues contributes to the pathogenesis of diabetic nephropathy High-glucose-inhibited ACE2 mRNA levels are restored by the TGF-β receptor inhibitor SB431542	[108]
Estrogen	Estrogen treatment decreases ACE2 mRNA levels in normal human bronchial epithelial cells The estrogen 17β-estradiol decreases renal ACE2 enzyme activity in the sex-chromosome-independent manner	[11, 12]
Miz1	ACE2 mRNA levels are increased in the MIZ1-deficient mice	[113]
HIF-1α	ACE2 mRNA levels are inhibited by HIF-1α	[114]
TNF-α	ACE2 mRNA levels may be decreased by TNF-α signaling in the ileum of Crohn’s disease parents	[117]

**Table 5:** Post-translational modification and regulation of ACE2 protein

Post-translational modification and regulation of ACE2 protein		
Name	Description	Ref.
Phosphorylation		
AMPK	AMPK mediates ACE2 Ser680 phosphorylation, inhibiting MDM2-mediated ACE2 ubiquitination	[116, 125]
CK1α	CK1α phosphorylates ACE2 (^3^SSS^5^) and induces the binding of E3 ligase SPOP to ACE2, leading to prevention of ACE2 protein from other E3 ligase-mediated protein degradation Infection of SARS-CoV-2 is attenuated by the CK1α inhibitor lenalidomide ACE2 protein levels are decreased by the CK1α inhibitor D4476, epiblastin A, and lenalidomide	[147]
MAP4K3 (GLK)	GLK phosphorylates ACE2 Ser776 and Ser783, leading to inhibition of UBR4-mediated ACE2 ubiquitination GLK expression is induced in the epithelial cells from COVID-19 patients ACE2 protein levels are decreased by the GLK inhibitor verteporfin	[28]
NUAK2	NUAK2 maintains ACE2 proteins on cell surface, resulting in enhancement of SARS-CoV-2 infection Surface ACE2 protein levels are decreased by the NUAK2 inhibitor WZ-4003	[149]
Ubiquitination/deubiquitination		
MDM2	MDM2 induces ACE2 ubiquitination at Lys788, resulting in proteasomal degradation of ACE2 protein MDM2-mediated ubiquitination is inhibited by AMPK-induced ACE2 phosphorylation MDM2 levels are induced in the lung tissues from IPAH patients ACE2 protein levels are increased by the MDM2 inhibitor JNJ-26854165 (JNJ-165)	[125]
NEDD4L	Ang II may decreases ACE2 protein levels through NEDD4L-mediated ubiquitination	[126]
Skp2	Skp2 induces ACE2 ubiquitination and degradation Skp2 expression is induced through the tobacco carcinogen BaP ACE2 protein levels are increased by inhibition Skp2 through the CDK4/6 inhibitor palbociclib	[127, 131]
UBR4	USBR4 induces ubiquitination of ACE2 protein at Lys26, Lys112, and Lys114, leading to proteasomal degradation of ACE2 protein UBR4-mediated ubiquitination is inhibited by GLK-induced ACE2 phosphorylation during SARS-CoV-2 infection	[28]
UCHL1	SARS-CoV-2 S protein stabilizes ACE2 protein through UCHL1-mediated deubiquitination, contributing to enhancement of SARS-CoV-2 infection ACE2 protein levels are decreased by the UCHL1 inhibitor LDN-57444	[29]
SPOP	The E3 ligase SPOP binds to CK1α-phosphorylated ACE2 at ^1^MSSSS^5^ residues, leading to prevention of ACE2 protein from other E3 ligase-mediated protein degradation	[147]
USP50	USP50 is a deubiquitinase that removes ubiquitins from its target proteins USP50 reduces ACE2 Lys48-linked ubiquitination at Lys788 residue and enhances ACE2 protein stability Vit C inhibits USP50-mediated ACE2 deubiquitination, resulting in downregulation of ACE2 proteins and attenuation of SARS-CoV-2 infection	[153]
Exosomal regulation		
MAP4K3 (GLK)	GLK induces exosomal ACE2 in COVID-19 patients GLK expression is induced in the lung epithelial cells from COVID-19 patient	[28]
Ectodomain shedding		
ADAM17	ADAM17 induces ACE2 ectodomain shedding and turns ACE2 into the catalytic activity-retaining soluble form ADAM17 cleavage sites on the ACE2 protein are at the region between ACE2 Ser716 and Ile741 residue or individual Ser709, Leu584, Arg652, Lys657, and Lys659 The soluble ACE2 protein levels are increased in the urine of human type 1 or type 2 diabetes patients and in the peripheral blood of myocardial infarction-induced heart failure patients Soluble ACE2 binds to SARS-CoV-2 S protein, facilitating cell entry of SARS-CoV-2 through receptor-mediated endocytosis Soluble ACE2 protein levels are decreased by the metalloproteinase inhibitor GM6001, as well as ADAM17 inhibitors GW280264X, DPC333, and TIMP-3	[135–139, 143, 162]
Lysosomal degradation		
AP2	High concentration of SARS-CoV-2 S protein induces ACE2 lysosomal degradation through AP2/clatherin-mediated endocytosis	[166]
SUMOylation		
PIAS4	The E3 ligase PIAS4 induces SUMO3 SUMOylation of ACE2 protein, preventing ACE2 autophagy ACE2 SUMO3 SUMOylation is mainly induced at Lys187	[169]
SENP3	SENP3-mediated deSUMOylation decreases ACE2 protein levels, contributing to attenuation of SARS-CoV-2 virus infection	[169]
Arginine methylation		
PRMT5	PRMT5 induces ACE2 protein methylation at Arg671, facilitating SARS-CoV-2 infection Infection of SARS-CoV-2 is attenuated by the PRMT5 inhibitor GSK3326595	[170]

**Fig. 6:**
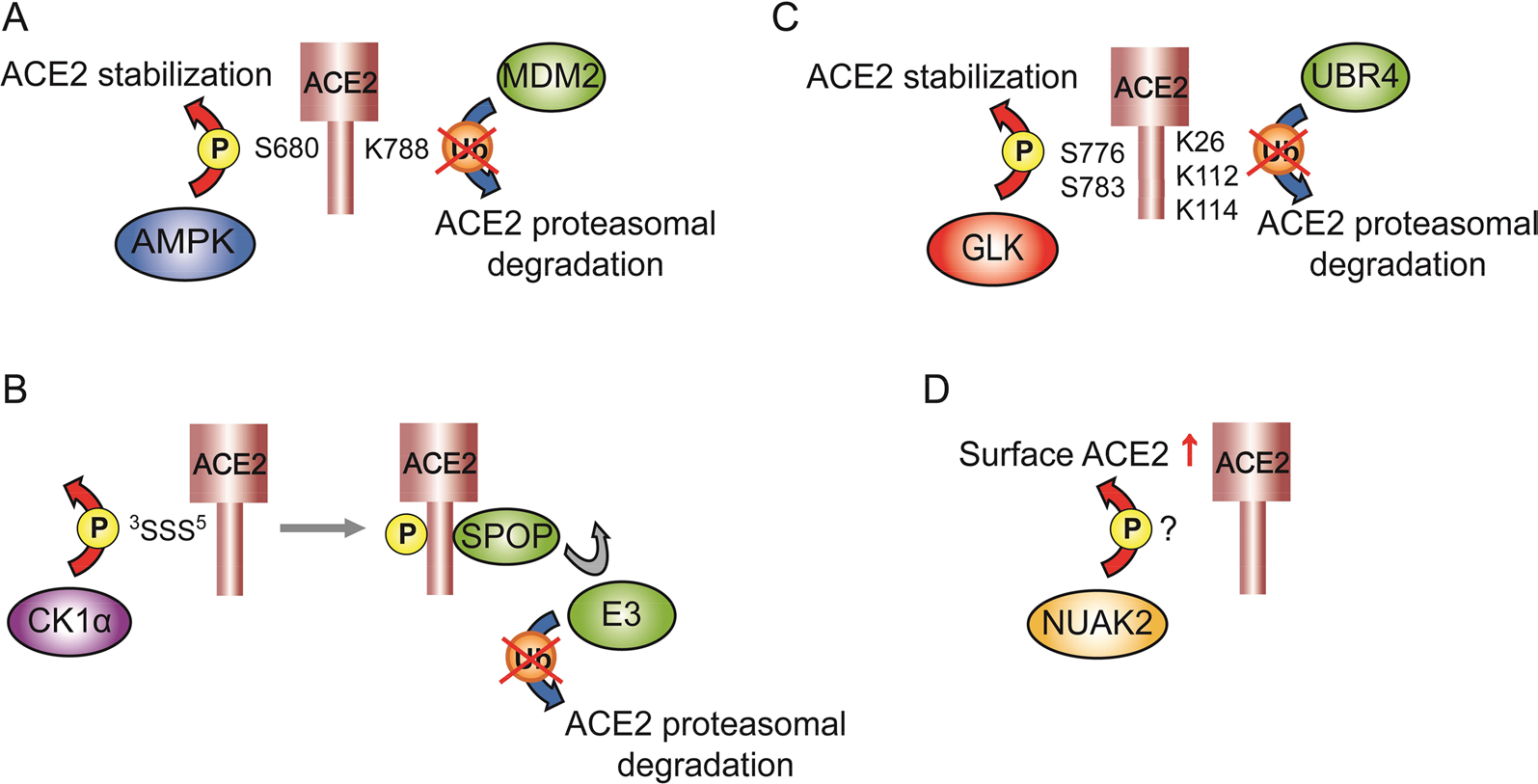
Phosphorylation-mediated deubiquitination and stabilization of ACE2 protein. **A** ACE2 Ser680 phosphorylation by AMPK reduces MDM2-mediated ACE2 Lys788 ubiquitination, resulting in ACE2 protein stabilization. **B** ACE2 Ser776 and Ser783 phosphorylation by GLK (MAP4K3) reduces UBR4-mediated ACE2 Lys26, Lys112, and Lys114 ubiquitination, resulting in enhancement of ACE2 stabilization. **C** CK1α phosphorylates ACE2 in the ^3^SSS^5^ region, inducing the interaction between ACE2 and SPOP. Binding of SPOP prevents ACE2 ubiquitination from other E3 ligase(s), leading to protection of ACE2 protein from proteasomal degradation. a**D** The induction of surface ACE protein levels may be mediated by phosphorylation of the kinase NUAK2 at unidentified ACE2 residues. Red arrows denote enhancement of ACE2 stability; blue arrows denote ACE2 downregulation through proteasomal degradation

In contrast to the benefits of ACE2 enhancement in chronic diseases, induction of the virus-entry receptor ACE2 would bring risks of enhancing virus infection. Thus, development of therapeutic drugs that block ACE2 induction but maintain basal ACE2 levels during SARS-CoV-2 infection may be beneficial for treatment. Interestingly, post-translational modification and regulation of ACE2 protein in chronic diseases are different from those of ACE2 protein in COVID-19. AMPK phosphorylates ACE2 at Ser680 residue, resulting in reduction of MDM2-mediated ACE2 Lys788 ubiquitination and degradation [116, 125] (Fig. 6). AMPK inactivation and ACE2 downregulation occur in the lung tissues of human pulmonary arterial hypertension patients [116]. CK1α also phosphorylates ACE2 protein at Ser3, Ser4, Ser5 residues and induces the interaction of ACE2 with the E3 ligase SPOP, which protects ACE2 protein from proteasomal degradation by unidentified E3 ligases other than MDM2 [147] (Fig. 6). Tobacco smoke-induced Skp2 also mediates ACE2 ubiquitination and proteasomal degradation in lung epithelial cells [127]. The putative ACE2 E3 ligase competing with SPOP could be either Skp2 or UBR4 (Fig. 6). During SARS-CoV-2 infection, the spike (S) protein of SARS-CoV-2 induces GLK expression in the respiratory tract epithelial cells of human COVID-19 patients [28]. GLK phosphorylates ACE2 at Ser776 and Ser783, preventing ACE2 protein from UBR4-mediated Lys26/112/144 ubiquitination and subsequent proteasomal degradation [28] (Fig. 5). The GLK-induced ACE2 Ser776/783 phosphorylation, but not AMPK-induced ACE2 Ser680 phosphorylation, is detected in serum exosomes of COVID-19 patients [28]. Moreover, the deubiquitinase UCHL1-mediated ACE2 deubiquitination [29], the arginine methyltransferase PRMT5-mediated ACE2 methylation [170], or the E3 SUMO-protein ligase PIAS4-mediated ACE2 SUMOylation [169] enhances ACE2 protein levels, facilitating SARS-CoV-2 infection. Conversely, treatment of the GLK inhibitor verteporfin attenuates ACE2 protein levels and SARS-CoV-2 pseudovirus infection in an ACE2-humanized mouse model and lung epithelial cells in vitro [28]. The UCHL1 inhibitor LDN-57444 decreases ACE2 protein levels and reduces live SARS-CoV-2 infection to cultured human epithelial cells [29]; the PRMT5 inhibitor GSK3326595 also suppresses SARS-CoV-2 pseudovirus infection in lung epithelial cells in vitro [170]. Furthermore, treatment of SUMOylation inhibitor decreases ACE2 protein levels and attenuates SARS-CoV-2 infection in vivo and in vitro [169]. Collectively, inhibition of GLK, UCHL1, PRMT5, or PIAS4 may suppress ACE2 overexpression in lung epithelial cells and attenuate COVID-19 symptoms without affecting basal ACE2 levels.

The binding of soluble form ACE2 (sACE2) to SARS-CoV-2 S protein facilitates cell entry of SARS-CoV-2 virus through endocytosis of the sACE2-S-vasopressin complex. In contrast, sACE2 protein may be used as a decoy to neutralize SARS-CoV-2 virus particles and attenuate virus infection [180]; however, only pre-mixed the decoy sACE2 with SARS-CoV-2 pseudovirus could achieve neutralizing effects [180]. Exosomal full-length ACE2 is induced in the serum of COVID-19 patients [28]; exosomal ACE2 is transported to recipient epithelial cells and enhances the susceptibility of SARS-CoV-2 infection [28]. In addition, the binding of soluble form ACE2 (sACE2) to SARS-CoV-2 spike (S) protein facilitates cell entry of SARS-CoV-2 virus through endocytosis of the sACE2-S-vasopressin complex [162]. ACE2-containing exosomes and sACE2 proteins have been tested as a decoy to attenuate SARS-CoV-2 infection [163, 180]; however, only simultaneous co-treatment or pre-mixed the decoy (sACE2) with SARS-CoV-2 pseudovirus could achieve neutralizing effects [180]. Further modification of the decoy ACE2 protein may generate a novel decoy that shows a higher binding affinity to spike (S) protein but lower endocytic ability. For example, ACE2 Asn90/322 N-glycosylation decreases its binding to the spike (S) protein [174, 176, 179]; conversely, ACE2 N322Q/N90A glycosylation-deficient mutant strongly increases its binding to the spike (S) protein [174, 176]. In addition, deficiency of the vasopressin receptor AVPR1B drastically reduces SARS-CoV-2 infection in human HK-2 cells [162], suggesting that loss of vasopressin-binding motif on sACE2 protein may block the endocytosis of sACE2-S complex. For example, the sACE2 protein with N322Q/N90A glycosylation-deficient mutation plus vasopressin-binding motif mutation could be used as a useful decoy molecule for the prevention of SARS-CoV-2 infection.

In summary, the enhancement and reduction of ACE2 will be beneficial for the prevention/treatment of chronic disease and COVID-19, respectively. Post-translational modifications of ACE2 protein in chronic diseases are different from those in COVID-19; therefore, regulation of ACE2 protein modification will help develop the prevention or treatment for COVID-19 without the induction of chronic disease risks.

## Data Availability

Not applicable.

## Abbreviations

ACE1: Angiotensin-converting enzyme 1
ACE2: Angiotensin-converting enzyme 2
ADAM17: A disintegrin and metalloprotease 17
AGT: Angiotensinogen
AICAR: 5-Amino-4-imidazolecarboxamide riboside
AMPK: AMP-activated protein kinase
Ang-(1–7): Angiotensin 1 to 7
Angiotensin II: Ang II
AP2: Adaptor protein complex 2
APJ: Apelin receptor
AT1R: Ang II type 1 receptor
AT2R: Ang II type 2 receptor
BaP: Benzo(a)pyrene
Brg1: Brahma-related gene-1
CD: Crohn’s disease
CHI3L1: Chitinase 3-like-1
ChIP: Chromatin immunoprecipitation
ChIP-seq: Chromatin immunoprecipitation sequencing
CK1α: Casein kinase 1α
COVID-19: Severe acute respiratory syndrome coronavirus 2 infection
CSE: Cigarette smoke extract
DYRK1A: Dual specificity tyrosine phosphorylation regulated kinase 1A
E2: 17β-Estradiol
ELA: Elabela
ERRα: Estrogen-related receptor α
ESCs: Embryonic stem cells
EZH2: Enhancer of zeste homologue 2
FoxM1: Forkhead box M1
GATA6: GATA binding protein 6
GEO: Gene Expression Omnibus
GLK: MAP4K3
H3-ac: Histone 3 acetylation
H3K4me1: Monomethylation of histone 3 on lysine 4
H3K4me3: Trimethylation of histone 3 on lysine 4
H3K27ac: Monoacetylation of histone 3 on lysine 27
H3K27me3: Trimethylation of histone 3 on lysine 27
HASMC: Human aortic smooth muscle cells
HCD: High cholesterol diet
HIF-1α: Hypoxia-inducible factor-1α
HNF1α: Hepatocyte nuclear factor 1α
HNF1β: Hepatocyte nuclear factor 1β
hPAMCs: Human pulmonary artery smooth muscle cells
IFNs: Interferons
IL-1: Interleukin 1
IL-1β: Interleukin 1β
IL-6: Interleukin 6
IL-13: Interleukin 13
IL-17A: Interleukin 17A
IPAH: Idiopathic pulmonary arterial hypertension
MD: Molecular dynamics
miR-125b: MicroRNA 125b
miR-200c-3p: MicroRNA 200c from the 3′ end of precursor microRNA hairpin
miR-421: MicroRNA 421
miR-483-3p: MicroRNA 483 from the 3′ end of precursor microRNA hairpin
Miz1: Myc-interacting zinc finger protein-1
MODY: Maturity-onset diabetes of the young
Nrf2: Nuclear factor erythroid 2-related factor 2
NUAK2: NUAK family kinase 2
PAH: Pulmonary arterial hypertension
PAP: Pulmonary arterial pressure
PRMT5: Protein arginine methyltransferase 5
RAS: Renin-angiotensin system
RASMC: Rat aortic smooth muscle cells
ROS: Reactive oxygen species
RPTCs: Renal proximal tubular cells
S protein: Spike protein
sACE2: Soluble ACE2
SARS-CoV: Severe acute respiratory syndrome coronavirus
SARS-CoV-2: Severe acute respiratory syndrome coronavirus 2
scRNA-seq: Single-cell RNA sequencing
SENP3: SUMO specific peptidase 3
SHRs: Spontaneously hypertensive rats
SIRT1: Silent information regulator T1
STAT3: Signal transducer and activator of transcription 3
SUMO3: Ubiquitin-like modifier 3
T1D: Type 1 diabetes
T2D: Type 2 diabetes
TAC: Transaortic constriction
TGF-β: Transforming growth factor-β
TNF-α: Tumor necrosis factor- α
TOLLIP: Toll-interacting protein
Treg: Regulatory T cells
UC: Ulcerative colitis
UCHL1: Ubiquitin carboxyl-terminal hydrolase L1
USP4: Ubiquitin specific proteases 4
USP50: Ubiquitin‐specific peptidase 50
Vit C: Vitamin C
VSMC: Vascular smooth muscle cell

## References

[1] Donoghue M, Hsieh F, Baronas E, Godbout K, Gosselin M, Stagliano N, Donovan M, Woolf B, Robison K, Jeyaseelan R (2000). A novel angiotensin-converting enzyme-related carboxypeptidase (ACE2) converts angiotensin I to angiotensin 1–9. Circ Res.

[2] Tipnis SR, Hooper NM, Hyde R, Karran E, Christie G, Turner AJ (2000). A human homolog of angiotensin-converting enzyme. Cloning and functional expression as a captopril-insensitive carboxypeptidase. J Biol Chem.

[3] Wiese O, Zemlin AE, Pillay TS (2021). Molecules in pathogenesis: angiotensin converting enzyme 2 (ACE2). J Clin Pathol.

[4] Bader M (2013). ACE2, angiotensin-(1–7), and Mas: the other side of the coin. Pflugers Arch.

[5] Pang XC, Zhang HX, Zhang Z, Rinkiko S, Cui YM, Zhu YZ (2020). The two-way switch role of ACE2 in the treatment of novel coronavirus pneumonia and underlying comorbidities. Molecules.

[6] Li W, Moore MJ, Vasilieva N, Sui J, Wong SK, Berne MA, Somasundaran M, Sullivan JL, Luzuriaga K, Greenough TC (2003). Angiotensin-converting enzyme 2 is a functional receptor for the SARS coronavirus. Nature.

[7] Hoffmann M, Kleine-Weber H, Schroeder S, Kruger N, Herrler T, Erichsen S, Schiergens TS, Herrler G, Wu NH, Nitsche A (2020). SARS-CoV-2 cell entry depends on ACE2 and TMPRSS2 and is blocked by a clinically proven protease inhibitor. Cell.

[8] Gkogkou E, Barnasas G, Vougas K, Trougakos IP (2020). Expression profiling meta-analysis of ACE2 and TMPRSS2, the putative anti-inflammatory receptor and priming protease of SARS-CoV-2 in human cells, and identification of putative modulators. Redox Biol.

[9] Hikmet F, Mear L, Edvinsson A, Micke P, Uhlen M, Lindskog C (2020). The protein expression profile of ACE2 in human tissues. Mol Syst Biol.

[10] Kamle S, Ma B, He CH, Akosman B, Zhou Y, Lee CM, El-Deiry WS, Huntington K, Liang O, Machan JT (2021). Chitinase 3-like-1 is a therapeutic target that mediates the effects of aging in COVID-19. JCI Insight.

[11] Liu J, Ji H, Zheng W, Wu X, Zhu JJ, Arnold AP, Sandberg K (2010). Sex differences in renal angiotensin converting enzyme 2 (ACE2) activity are 17β-oestradiol-dependent and sex chromosome-independent. Biol Sex Differ.

[12] Stelzig KE, Canepa-Escaro F, Schiliro M, Berdnikovs S, Prakash YS, Chiarella SE (2020). Estrogen regulates the expression of SARS-CoV-2 receptor ACE2 in differentiated airway epithelial cells. Am J Physiol Lung Cell Mol Physiol.

[13] Bindom SM, Hans CP, Xia H, Boulares AH, Lazartigues E (2010). Angiotensin I-converting enzyme type 2 (ACE2) gene therapy improves glycemic control in diabetic mice. Diabetes.

[14] Ye M, Wysocki J, Naaz P, Salabat MR, LaPointe MS, Batlle D (2004). Increased ACE 2 and decreased ACE protein in renal tubules from diabetic mice: a renoprotective combination?. Hypertension.

[15] Wysocki J, Ye M, Soler MJ, Gurley SB, Xiao HD, Bernstein KE, Coffman TM, Chen S, Batlle D (2006). ACE and ACE2 activity in diabetic mice. Diabetes.

[16] Ye M, Wysocki J, William J, Soler MJ, Cokic I, Batlle D (2006). Glomerular localization and expression of Angiotensin-converting enzyme 2 and Angiotensin-converting enzyme: implications for albuminuria in diabetes. J Am Soc Nephrol.

[17] Tikellis C, Wookey PJ, Candido R, Andrikopoulos S, Thomas MC, Cooper ME (2004). Improved islet morphology after blockade of the renin- angiotensin system in the ZDF rat. Diabetes.

[18] Moon JY, Jeong KH, Lee SH, Lee TW, Ihm CG, Lim SJ (2008). Renal ACE and ACE2 expression in early diabetic rats. Nephron Exp Nephrol.

[19] Chodavarapu H, Grobe N, Somineni HK, Salem ES, Madhu M, Elased KM (2013). Rosiglitazone treatment of type 2 diabetic db/db mice attenuates urinary albumin and angiotensin converting enzyme 2 excretion. PLoS ONE.

[20] Salem ES, Grobe N, Elased KM (2014). Insulin treatment attenuates renal ADAM17 and ACE2 shedding in diabetic Akita mice. Am J Physiol Renal Physiol.

[21] Tikellis C, Johnston CI, Forbes JM, Burns WC, Burrell LM, Risvanis J, Cooper ME (2003). Characterization of renal angiotensin-converting enzyme 2 in diabetic nephropathy. Hypertension.

[22] Leehey DJ, Singh AK, Bast JP, Sethupathi P, Singh R (2008). Glomerular renin angiotensin system in streptozotocin diabetic and Zucker diabetic fatty rats. Transl Res.

[23] Tikellis C, Pickering R, Tsorotes D, Du XJ, Kiriazis H, Nguyen-Huu TP, Head GA, Cooper ME, Thomas MC (2012). Interaction of diabetes and ACE2 in the pathogenesis of cardiovascular disease in experimental diabetes. Clin Sci.

[24] Zhao S, Ghosh A, Lo CS, Chenier I, Scholey JW, Filep JG, Ingelfinger JR, Zhang SL, Chan JSD (2018). Nrf2 deficiency upregulates intrarenal angiotensin-converting enzyme-2 and angiotensin 1–7 receptor expression and attenuates hypertension and nephropathy in diabetic mice. Endocrinology.

[25] Beacon TH, Delcuve GP, Davie JR (2021). Epigenetic regulation of ACE2, the receptor of the SARS-CoV-2 virus(1). Genome.

[26] Bunyavanich S, Do A, Vicencio A (2020). Nasal gene expression of angiotensin-converting enzyme 2 in children and adults. JAMA.

[27] Reindl-Schwaighofer R, Hodlmoser S, Eskandary F, Poglitsch M, Bonderman D, Strassl R, Aberle JH, Oberbauer R, Zoufaly A, Hecking M (2021). ACE2 elevation in severe COVID-19. Am J Respir Crit Care Med.

[28] Chuang HC, Hsueh CH, Hsu PM, Huang RH, Tsai CY, Chung NH, Chow YH, Tan TH (2022). SARS-CoV-2 spike protein enhances MAP4K3/GLK-induced ACE2 stability in COVID-19. EMBO Mol Med.

[29] Bednash JS, Johns F, Farkas D, Elhance A, Adair J, Cress K, Yount JS, Kenney AD, Londino JD, Mallampalli RK (2023). Inhibiting the deubiquitinase UCHL1 reduces SARS-CoV-2 viral uptake by ACE2. Am J Respir Cell Mol Biol.

[30] Kuan TC, Yang TH, Wen CH, Chen MY, Lee IL, Lin CS (2011). Identifying the regulatory element for human angiotensin-converting enzyme 2 (ACE2) expression in human cardiofibroblasts. Peptides.

[31] Nunes-Santos CJ, Kuehn HS, Rosenzweig SD (2020). IKAROS family zinc finger 1-associated diseases in primary immunodeficiency patients. Immunol Allergy Clin North Am.

[32] Yamagata K (2003). Regulation of pancreatic beta-cell function by the HNF transcription network: lessons from maturity-onset diabetes of the young (MODY). Endocr J.

[33] Senkel S, Lucas B, Klein-Hitpass L, Ryffel GU (2005). Identification of target genes of the transcription factor HNF1β and HNF1α in a human embryonic kidney cell line. Biochim Biophys Acta.

[34] Pedersen KB, Chhabra KH, Nguyen VK, Xia H, Lazartigues E (2013). The transcription factor HNF1α induces expression of angiotensin-converting enzyme 2 (ACE2) in pancreatic islets from evolutionarily conserved promoter motifs. Biochim Biophys Acta.

[35] Niehof M, Borlak J (2011). HNF4α dysfunction as a molecular rational for cyclosporine induced hypertension. PLoS ONE.

[36] Israeli M, Finkel Y, Yahalom-Ronen Y, Paran N, Chitlaru T, Israeli O, Cohen-Gihon I, Aftalion M, Falach R, Rotem S (2022). Genome-wide CRISPR screens identify GATA6 as a proviral host factor for SARS-CoV-2 via modulation of ACE2. Nat Commun.

[37] Liang LJ, Wang D, Yu H, Wang J, Zhang H, Sun BB, Yang FY, Wang Z, Xie DW, Feng RE (2022). Transcriptional regulation and small compound targeting of ACE2 in lung epithelial cells. Acta Pharmacol Sin.

[38] Mokuda S, Tokunaga T, Masumoto J, Sugiyama E (2020). Angiotensin-converting enzyme 2, a SARS-CoV-2 receptor, is upregulated by interleukin 6 through STAT3 signaling in synovial tissues. J Rheumatol.

[39] Canto C, Auwerx J (2009). Caloric restriction, SIRT1 and longevity. Trends Endocrinol Metab.

[40] Wang Y, Liang Y, Vanhoutte PM (2011). SIRT1 and AMPK in regulating mammalian senescence: a critical review and a working model. FEBS Lett.

[41] Hardie DG, Alessi DR (2013). LKB1 and AMPK and the cancer-metabolism link—ten years after. BMC Biol.

[42] Bonora M, Patergnani S, Rimessi A, De Marchi E, Suski JM, Bononi A, Giorgi C, Marchi S, Missiroli S, Poletti F (2012). ATP synthesis and storage. Purinergic Signal.

[43] Ruderman NB, Xu XJ, Nelson L, Cacicedo JM, Saha AK, Lan F, Ido Y (2010). AMPK and SIRT1: a long-standing partnership?. Am J Physiol Endocrinol Metab.

[44] Lan F, Cacicedo JM, Ruderman N, Ido Y (2008). SIRT1 modulation of the acetylation status, cytosolic localization, and activity of LKB1. Possible role in AMP-activated protein kinase activation. J Biol Chem.

[45] Coughlan KA, Valentine RJ, Ruderman NB, Saha AK (2014). AMPK activation: a therapeutic target for type 2 diabetes?. Diabetes Metab Syndr Obes Targets Ther.

[46] Fulco M, Cen Y, Zhao P, Hoffman EP, McBurney MW, Sauve AA, Sartorelli V (2008). Glucose restriction inhibits skeletal myoblast differentiation by activating SIRT1 through AMPK-mediated regulation of Nampt. Dev Cell.

[47] Canto C, Auwerx J (2009). PGC-1alpha, SIRT1 and AMPK, an energy sensing network that controls energy expenditure. Curr Opin Lipidol.

[48] Canto C, Gerhart-Hines Z, Feige JN, Lagouge M, Noriega L, Milne JC, Elliott PJ, Puigserver P, Auwerx J (2009). AMPK regulates energy expenditure by modulating NAD^+^ metabolism and SIRT1 activity. Nature.

[49] Clarke NE, Belyaev ND, Lambert DW, Turner AJ (2014). Epigenetic regulation of angiotensin-converting enzyme 2 (ACE2) by SIRT1 under conditions of cell energy stress. Clin Sci.

[50] Di Cerbo V, Mohn F, Ryan DP, Montellier E, Kacem S, Tropberger P, Kallis E, Holzner M, Hoerner L, Feldmann A (2014). Acetylation of histone H3 at lysine 64 regulates nucleosome dynamics and facilitates transcription. Elife.

[51] Kim J, Lee J, Lee TH (2015). Lysine acetylation facilitates spontaneous DNA dynamics in the nucleosome. J Phys Chem B.

[52] Tikoo K, Patel G, Kumar S, Karpe PA, Sanghavi M, Malek V, Srinivasan K (2015). Tissue specific up regulation of ACE2 in rabbit model of atherosclerosis by atorvastatin: role of epigenetic histone modifications. Biochem Pharmacol.

[53] Pinto BGG, Oliveira AER, Singh Y, Jimenez L, Goncalves ANA, Ogava RLT, Creighton R, Schatzmann Peron JP, Nakaya HI (2020). ACE2 expression is increased in the lungs of patients with comorbidities associated with severe COVID-19. J Infect Dis.

[54] Aranda S, Laguna A, de la Luna S (2011). DYRK family of protein kinases: evolutionary relationships, biochemical properties, and functional roles. FASEB J.

[55] Soppa U, Becker W (2015). DYRK protein kinases. Curr Biol.

[56] Garcia-Cerro S, Martinez P, Vidal V, Corrales A, Florez J, Vidal R, Rueda N, Arbones ML, Martinez-Cue C (2014). Overexpression of Dyrk1A is implicated in several cognitive, electrophysiological and neuromorphological alterations found in a mouse model of Down syndrome. PLoS ONE.

[57] Park J, Yang EJ, Yoon JH, Chung KC (2007). Dyrk1A overexpression in immortalized hippocampal cells produces the neuropathological features of Down syndrome. Mol Cell Neurosci.

[58] Clift AK, Coupland CAC, Keogh RH, Hemingway H, Hippisley-Cox J (2021). COVID-19 mortality risk in Down syndrome: results from a cohort study of 8 million adults. Ann Intern Med.

[59] De Toma I, Dierssen M (2021). Network analysis of Down syndrome and SARS-CoV-2 identifies risk and protective factors for COVID-19. Sci Rep.

[60] Illouz T, Biragyn A, Frenkel-Morgenstern M, Weissberg O, Gorohovski A, Merzon E, Green I, Iulita F, Flores-Aguilar L, Dierssen M (2021). Specific susceptibility to COVID-19 in adults with Down syndrome. Neuromolecular Med.

[61] Malle L, Gao C, Hur C, Truong HQ, Bouvier NM, Percha B, Kong XF, Bogunovic D (2021). Individuals with Down syndrome hospitalized with COVID-19 have more severe disease. Genet Med.

[62] Strine MS, Cai WL, Wei J, Alfajaro MM, Filler RB, Biering SB, Sarnik S, Chow RD, Patil A, Cervantes KS (2023). DYRK1A promotes viral entry of highly pathogenic human coronaviruses in a kinase-independent manner. PLoS Biol.

[63] Bailey CC, Zhong G, Huang IC, Farzan M (2014). IFITM-family proteins: the cell’s first line of antiviral defense. Annu Rev Virol.

[64] Sajuthi SP, DeFord P, Li Y, Jackson ND, Montgomery MT, Everman JL, Rios CL, Pruesse E, Nolin JD, Plender EG (2020). Type 2 and interferon inflammation regulate SARS-CoV-2 entry factor expression in the airway epithelium. Nat Commun.

[65] Ziegler CGK, Allon SJ, Nyquist SK, Mbano IM, Miao VN, Tzouanas CN, Cao Y, Yousif AS, Bals J, Hauser BM (2020). SARS-CoV-2 receptor ACE2 is an interferon-stimulated gene in human airway epithelial cells and is detected in specific cell subsets across tissues. Cell.

[66] Hou YJ, Okuda K, Edwards CE, Martinez DR, Asakura T, Dinnon KH, Kato T, Lee RE, Yount BL, Mascenik TM (2020). SARS-CoV-2 reverse genetics reveals a variable infection gradient in the respiratory tract. Cell.

[67] Matys V, Fricke E, Geffers R, Gossling E, Haubrock M, Hehl R, Hornischer K, Karas D, Kel AE, Kel-Margoulis OV (2003). TRANSFAC: transcriptional regulation, from patterns to profiles. Nucleic Acids Res.

[68] Rusinova I, Forster S, Yu S, Kannan A, Masse M, Cumming H, Chapman R, Hertzog PJ (2013). Interferome v2.0: an updated database of annotated interferon-regulated genes. Nucleic Acids Res.

[69] Wang J, Zhuang J, Iyer S, Lin XY, Greven MC, Kim BH, Moore J, Pierce BG, Dong X, Virgil D (2013). Factorbook.org: a Wiki-based database for transcription factor-binding data generated by the ENCODE consortium. Nucleic Acids Res.

[70] Sherman EJ, Mirabelli C, Tang VT, Khan TG, Leix K, Kennedy AA, Graham SE, Willer CJ, Tai AW, Sexton JZ (2022). Identification of cell type specific ACE2 modifiers by CRISPR screening. PLoS Pathog.

[71] Yang J, Feng X, Zhou Q, Cheng W, Shang C, Han P, Lin CH, Chen HS, Quertermous T, Chang CP (2016). Pathological ACE2-to-ACE enzyme switch in the stressed heart is transcriptionally controlled by the endothelial Brg1-FoxM1 complex. Proc Natl Acad Sci USA.

[72] Hang CT, Yang J, Han P, Cheng HL, Shang C, Ashley E, Zhou B, Chang CP (2010). Chromatin regulation by Brg1 underlies heart muscle development and disease. Nature.

[73] Huss JM, Garbacz WG, Xie W (2015). Constitutive activities of estrogen-related receptors: transcriptional regulation of metabolism by the ERR pathways in health and disease. Biochim Biophys Acta.

[74] Tremblay AM, Dufour CR, Ghahremani M, Reudelhuber TL, Giguere V (2010). Physiological genomics identifies estrogen-related receptor α as a regulator of renal sodium and potassium homeostasis and the renin-angiotensin pathway. Mol Endocrinol.

[75] Vakoc CR, Mandat SA, Olenchock BA, Blobel GA (2005). Histone H3 lysine 9 methylation and HP1γ are associated with transcription elongation through mammalian chromatin. Mol Cell.

[76] Pan MR, Hsu MC, Chen LT, Hung WC (2018). Orchestration of H3K27 methylation: mechanisms and therapeutic implication. Cell Mol Life Sci.

[77] Li Y, Li H, Zhou L (2020). EZH2-mediated H3K27me3 inhibits ACE2 expression. Biochem Biophys Res Commun.

[78] Szostak J, Goracy A, Durys D, Dec P, Modrzejewski A, Pawlik A (2023). The role of microRNA in the pathogenesis of diabetic nephropathy. Int J Mol Sci.

[79] Huang YF, Zhang Y, Liu CX, Huang J, Ding GH (2016). microRNA-125b contributes to high glucose-induced reactive oxygen species generation and apoptosis in HK-2 renal tubular epithelial cells by targeting angiotensin-converting enzyme 2. Eur Rev Med Pharmacol Sci.

[80] Liu CX, Hu Q, Wang Y, Zhang W, Ma ZY, Feng JB, Wang R, Wang XP, Dong B, Gao F (2011). Angiotensin-converting enzyme (ACE) 2 overexpression ameliorates glomerular injury in a rat model of diabetic nephropathy: a comparison with ACE inhibition. Mol Med.

[81] Lambert DW, Lambert LA, Clarke NE, Hooper NM, Porter KE, Turner AJ (2014). Angiotensin-converting enzyme 2 is subject to post-transcriptional regulation by miR-421. Clin Sci.

[82] Liu Q, Du J, Yu X, Xu J, Huang F, Li X, Zhang C, Li X, Chang J, Shang D (2017). miRNA-200c-3p is crucial in acute respiratory distress syndrome. Cell Discov.

[83] Yang P, Gu H, Zhao Z, Wang W, Cao B, Lai C, Yang X, Zhang L, Duan Y, Zhang S (2014). Angiotensin-converting enzyme 2 (ACE2) mediates influenza H7N9 virus-induced acute lung injury. Sci Rep.

[84] Kemp JR, Unal H, Desnoyer R, Yue H, Bhatnagar A, Karnik SS (2014). Angiotensin II-regulated microRNA 483-3p directly targets multiple components of the renin-angiotensin system. J Mol Cell Cardiol.

[85] Sato T, Suzuki T, Watanabe H, Kadowaki A, Fukamizu A, Liu PP, Kimura A, Ito H, Penninger JM, Imai Y (2013). Apelin is a positive regulator of ACE2 in failing hearts. J Clin Invest.

[86] Sato T, Sato C, Kadowaki A, Watanabe H, Ho L, Ishida J, Yamaguchi T, Kimura A, Fukamizu A, Penninger JM (2017). ELABELA-APJ axis protects from pressure overload heart failure and angiotensin II-induced cardiac damage. Cardiovasc Res.

[87] Ma Z, Song JJ, Martin S, Yang XC, Zhong JC (2021). The Elabela-APJ axis: a promising therapeutic target for heart failure. Heart Fail Rev.

[88] Song JJ, Yang M, Liu Y, Song JW, Liu XY, Miao R, Zhang ZZ, Liu Y, Fan YF, Zhang Q (2021). Elabela prevents angiotensin II-induced apoptosis and inflammation in rat aortic adventitial fibroblasts via the activation of FGF21-ACE2 signaling. J Mol Histol.

[89] Vincent TL (2019). IL-1 in osteoarthritis: time for a critical review of the literature. F1000Res.

[90] Liu C, Lv XH, Li HX, Cao X, Zhang F, Wang L, Yu M, Yang JK (2012). Angiotensin-(1–7) suppresses oxidative stress and improves glucose uptake via Mas receptor in adipocytes. Acta Diabetol.

[91] Qian YR, Guo Y, Wan HY, Fan L, Feng Y, Ni L, Xiang Y, Li QY (2013). Angiotensin-converting enzyme 2 attenuates the metastasis of non-small cell lung cancer through inhibition of epithelial-mesenchymal transition. Oncol Rep.

[92] Ender SA, Dallmer A, Lassig F, Lendeckel U, Wolke C (2014). Expression and function of the ACE2/angiotensin(1–7)/Mas axis in osteosarcoma cell lines U-2 OS and MNNG-HOS. Mol Med Rep.

[93] Chupp GL, Lee CG, Jarjour N, Shim YM, Holm CT, He S, Dziura JD, Reed J, Coyle AJ, Kiener P (2007). A chitinase-like protein in the lung and circulation of patients with severe asthma. N Engl J Med.

[94] Ober C, Tan Z, Sun Y, Possick JD, Pan L, Nicolae R, Radford S, Parry RR, Heinzmann A, Deichmann KA (2008). Effect of variation in CHI3L1 on serum YKL-40 level, risk of asthma, and lung function. N Engl J Med.

[95] Kastrup J, Johansen JS, Winkel P, Hansen JF, Hildebrandt P, Jensen GB, Jespersen CM, Kjoller E, Kolmos HJ, Lind I (2009). High serum YKL-40 concentration is associated with cardiovascular and all-cause mortality in patients with stable coronary artery disease. Eur Heart J.

[96] Ahangari F, Sood A, Ma B, Takyar S, Schuyler M, Qualls C, Dela Cruz CS, Chupp GL, Lee CG, Elias JA (2015). Chitinase 3-like-1 regulates both visceral fat accumulation and asthma-like Th2 inflammation. Am J Respir Crit Care Med.

[97] Gomez JL, Crisafi GM, Holm CT, Meyers DA, Hawkins GA, Bleecker ER, Jarjour N, Cohn L, Chupp GL, Severe Asthma Research Program I (2015). Genetic variation in chitinase 3-like 1 (CHI3L1) contributes to asthma severity and airway expression of YKL-40. J Allergy Clin Immunol.

[98] Di Rosa M, Malaguarnera L (2016). Chitinase 3 like-1: an emerging molecule involved in diabetes and diabetic complications. Pathobiology.

[99] Schernthaner GH, Hobaus C, Brix J (2016). YKL-40 and its complex association with metabolic syndrome, obesity, and cardiovascular disease. Anatol J Cardiol.

[100] Li K, Chen Z, Qin Y, Wei YX (2019). Plasm YKL-40 levels are associated with hypertension in patients with obstructive sleep apnea. Biomed Res Int.

[101] Gu Q, Wang B, Zhang XF, Ma YP, Liu JD, Wang XZ (2014). Contribution of renin-angiotensin system to exercise-induced attenuation of aortic remodeling and improvement of endothelial function in spontaneously hypertensive rats. Cardiovasc Pathol.

[102] Fernandes T, Hashimoto NY, Magalhaes FC, Fernandes FB, Casarini DE, Carmona AK, Krieger JE, Phillips MI, Oliveira EM (2011). Aerobic exercise training-induced left ventricular hypertrophy involves regulatory MicroRNAs, decreased angiotensin-converting enzyme-angiotensin II, and synergistic regulation of angiotensin-converting enzyme 2-angiotensin (1–7). Hypertension.

[103] Koka V, Huang XR, Chung AC, Wang W, Truong LD, Lan HY (2008). Angiotensin II up-regulates angiotensin I-converting enzyme (ACE), but down-regulates ACE2 via the AT1-ERK/p38 MAP kinase pathway. Am J Pathol.

[104] Kimura H, Francisco D, Conway M, Martinez FD, Vercelli D, Polverino F, Billheimer D, Kraft M (2020). Type 2 inflammation modulates ACE2 and TMPRSS2 in airway epithelial cells. J Allergy Clin Immunol.

[105] Gandhi NA, Bennett BL, Graham NM, Pirozzi G, Stahl N, Yancopoulos GD (2016). Targeting key proximal drivers of type 2 inflammation in disease. Nat Rev Drug Discov.

[106] Shah IM, Mackay SP, McKay GA (2009). Therapeutic strategies in the treatment of diabetic nephropathy—a translational medicine approach. Curr Med Chem.

[107] Reich HN, Oudit GY, Penninger JM, Scholey JW, Herzenberg AM (2008). Decreased glomerular and tubular expression of ACE2 in patients with type 2 diabetes and kidney disease. Kidney Int.

[108] Chou CH, Chuang LY, Lu CY, Guh JY (2013). Interaction between TGF-β and ACE2-Ang-(1–7)-Mas pathway in high glucose-cultured NRK-52E cells. Mol Cell Endocrinol.

[109] Grasselli G, Zangrillo A, Zanella A, Antonelli M, Cabrini L, Castelli A, Cereda D, Coluccello A, Foti G, Fumagalli R (2020). Baseline characteristics and outcomes of 1591 patients infected with SARS-CoV-2 admitted to ICUs of the Lombardy region, Italy. JAMA.

[110] Guan WJ, Ni ZY, Hu Y, Liang WH, Ou CQ, He JX, Liu L, Shan H, Lei CL, Hui DSC (2020). Clinical characteristics of coronavirus disease 2019 in China. N Engl J Med.

[111] Bukowska A, Spiller L, Wolke C, Lendeckel U, Weinert S, Hoffmann J, Bornfleth P, Kutschka I, Gardemann A, Isermann B (2017). Protective regulation of the ACE2/ACE gene expression by estrogen in human atrial tissue from elderly men. Exp Biol Med.

[112] Do-Umehara HC, Chen C, Urich D, Zhou L, Qiu J, Jang S, Zander A, Baker MA, Eilers M, Sporn PH (2013). Suppression of inflammation and acute lung injury by Miz1 via repression of C/EBP-δ. Nat Immunol.

[113] Do-Umehara HC, Chen C, Zhang Q, Misharin AV, Abdala-Valencia H, Casalino-Matsuda SM, Reyfman PA, Anekalla KR, Gonzalez-Gonzalez FJ, Sala MA (2020). Epithelial cell-specific loss of function of Miz1 causes a spontaneous COPD-like phenotype and up-regulates Ace2 expression in mice. Sci Adv.

[114] Zhang R, Wu Y, Zhao M, Liu C, Zhou L, Shen S, Liao S, Yang K, Li Q, Wan H (2009). Role of HIF-1α in the regulation ACE and ACE2 expression in hypoxic human pulmonary artery smooth muscle cells. Am J Physiol Lung Cell Mol Physiol.

[115] Sharma V, Shaheen SS, Dixit D, Sen E (2012). Farnesyltransferase inhibitor manumycin targets IL1β-Ras-HIF-1α axis in tumor cells of diverse origin. Inflammation.

[116] Zhang J, Dong J, Martin M, He M, Gongol B, Marin TL, Chen L, Shi X, Yin Y, Shang F (2018). AMP-activated protein kinase phosphorylation of angiotensin-converting enzyme 2 in endothelium mitigates pulmonary hypertension. Am J Respir Crit Care Med.

[117] Potdar AA, Dube S, Naito T, Li K, Botwin G, Haritunians T, Li D, Casero D, Yang S, Bilsborough J (2021). Altered intestinal ACE2 levels are associated with inflammation, severe disease, and response to anti-cytokine therapy in inflammatory bowel disease. Gastroenterology.

[118] Onabajo OO, Banday AR, Stanifer ML, Yan W, Obajemu A, Santer DM, Florez-Vargas O, Piontkivska H, Vargas JM, Ring TJ (2020). Interferons and viruses induce a novel truncated ACE2 isoform and not the full-length SARS-CoV-2 receptor. Nat Genet.

[119] Blume C, Jackson CL, Spalluto CM, Legebeke J, Nazlamova L, Conforti F, Perotin JM, Frank M, Butler J, Crispin M (2021). A novel ACE2 isoform is expressed in human respiratory epithelia and is upregulated in response to interferons and RNA respiratory virus infection. Nat Genet.

[120] Jiang F, Yang J, Zhang Y, Dong M, Wang S, Zhang Q, Liu FF, Zhang K, Zhang C (2014). Angiotensin-converting enzyme 2 and angiotensin 1–7: novel therapeutic targets. Nat Rev Cardiol.

[121] Wang XX, Wang XL, Tong MM, Gan L, Chen H, Wu SS, Chen JX, Li RL, Wu Y, Zhang HY (2016). SIRT6 protects cardiomyocytes against ischemia/reperfusion injury by augmenting FoxO3α-dependent antioxidant defense mechanisms. Basic Res Cardiol.

[122] Zhang ZZ, Cheng YW, Jin HY, Chang Q, Shang QH, Xu YL, Chen LX, Xu R, Song B, Zhong JC (2017). The sirtuin 6 prevents angiotensin II-mediated myocardial fibrosis and injury by targeting AMPK-ACE2 signaling. Oncotarget.

[123] Iwakuma T, Lozano G (2003). MDM2, an introduction. Mol Cancer Res.

[124] Shangary S, Wang S (2008). Targeting the MDM2-p53 interaction for cancer therapy. Clin Cancer Res.

[125] Shen H, Zhang J, Wang C, Jain PP, Xiong M, Shi X, Lei Y, Chen S, Yin Q, Thistlethwaite PA (2020). MDM2-mediated ubiquitination of angiotensin-converting enzyme 2 contributes to the development of pulmonary arterial hypertension. Circulation.

[126] Mohammed M, Ogunlade B, Elgazzaz M, Berdasco C, Lakkappa N, Ghita I, Guidry JJ, Sriramula S, Xu J, Restivo L (2023). Nedd4-2 upregulation is associated with ACE2 ubiquitination in hypertension. Cardiovasc Res.

[127] Wang G, Zhao Q, Zhang H, Liang F, Zhang C, Wang J, Chen Z, Wu R, Yu H, Sun B (2021). Degradation of SARS-CoV-2 receptor ACE2 by the E3 ubiquitin ligase Skp2 in lung epithelial cells. Front Med.

[128] Farsalinos K, Barbouni A, Niaura R (2020). Systematic review of the prevalence of current smoking among hospitalized COVID-19 patients in China: could nicotine be a therapeutic option?. Intern Emerg Med.

[129] Vardavas CI, Nikitara K (2020). COVID-19 and smoking: a systematic review of the evidence. Tob Induc Dis.

[130] Kitagawa K, Kotake Y, Kitagawa M (2009). Ubiquitin-mediated control of oncogene and tumor suppressor gene products. Cancer Sci.

[131] Xiao Y, Yan Y, Chang L, Ji H, Sun H, Song S, Feng K, Nuermaimaiti A, Lu Z, Wang L (2023). CDK4/6 inhibitor palbociclib promotes SARS-CoV-2 cell entry by down-regulating SKP2 dependent ACE2 degradation. Antivir Res.

[132] Organization WH. Smoking and COVID-19. https://www.who.int/news-room/commentaries/detail/smoking-and-covid-19. Assessed 2 May 2023.

[133] Hu B, Zhang D, Zhao K, Wang Y, Pei L, Fu Q, Ma X (2021). Spotlight on USP4: structure, function, and regulation. Front Cell Dev Biol.

[134] Wang J, Xiang Y, Yang SX, Zhang HM, Li H, Zong QB, Li LW, Zhao LL, Xia RH, Li C (2022). MIR99AHG inhibits EMT in pulmonary fibrosis via the miR-136-5p/USP4/ACE2 axis. J Transl Med.

[135] Lambert DW, Yarski M, Warner FJ, Thornhill P, Parkin ET, Smith AI, Hooper NM, Turner AJ (2005). Tumor necrosis factor-alpha convertase (ADAM17) mediates regulated ectodomain shedding of the severe-acute respiratory syndrome-coronavirus (SARS-CoV) receptor, angiotensin-converting enzyme-2 (ACE2). J Biol Chem.

[136] Jia HP, Look DC, Tan P, Shi L, Hickey M, Gakhar L, Chappell MC, Wohlford-Lenane C, McCray PB (2009). Ectodomain shedding of angiotensin converting enzyme 2 in human airway epithelia. Am J Physiol Lung Cell Mol Physiol.

[137] Grobe N, Di Fulvio M, Kashkari N, Chodavarapu H, Somineni HK, Singh R, Elased KM (2015). Functional and molecular evidence for expression of the renin angiotensin system and ADAM17-mediated ACE2 shedding in COS7 cells. Am J Physiol Cell Physiol.

[138] Lai ZW, Hanchapola I, Steer DL, Smith AI (2011). Angiotensin-converting enzyme 2 ectodomain shedding cleavage-site identification: determinants and constraints. Biochemistry.

[139] Heurich A, Hofmann-Winkler H, Gierer S, Liepold T, Jahn O, Pohlmann S (2014). TMPRSS2 and ADAM17 cleave ACE2 differentially and only proteolysis by TMPRSS2 augments entry driven by the severe acute respiratory syndrome coronavirus spike protein. J Virol.

[140] Park SE, Kim WJ, Park SW, Park JW, Lee N, Park CY, Youn BS (2013). High urinary ACE2 concentrations are associated with severity of glucose intolerance and microalbuminuria. Eur J Endocrinol.

[141] Burns KD, Lytvyn Y, Mahmud FH, Daneman D, Deda L, Dunger DB, Deanfield J, Dalton RN, Elia Y, Har R (2017). The relationship between urinary renin-angiotensin system markers, renal function, and blood pressure in adolescents with type 1 diabetes. Am J Physiol Renal Physiol.

[142] Gilbert A, Liu J, Cheng G, An C, Deo K, Gorret AM, Qin X (2019). A review of urinary angiotensin converting enzyme 2 in diabetes and diabetic nephropathy. Biochem Med.

[143] Chen Q, Li Y, Bie B, Zhao B, Zhang Y, Fang S, Li S, Zhang Y (2023). P38 MAPK activated ADAM17 mediates ACE2 shedding and promotes cardiac remodeling and heart failure after myocardial infarction. Cell Commun Signal.

[144] Jiang Q, Zheng N, Bu L, Zhang X, Zhang X, Wu Y, Su Y, Wang L, Zhang X, Ren S (2021). SPOP-mediated ubiquitination and degradation of PDK1 suppresses AKT kinase activity and oncogenic functions. Mol Cancer.

[145] Su S, Chen J, Jiang Y, Wang Y, Vital T, Zhang J, Laggner C, Nguyen KT, Zhu Z, Prevatte AW (2021). SPOP and OTUD7A control EWS-FLI1 protein stability to govern ewing sarcoma growth. Adv Sci.

[146] Zhuang M, Calabrese MF, Liu J, Waddell MB, Nourse A, Hammel M, Miller DJ, Walden H, Duda DM, Seyedin SN (2009). Structures of SPOP-substrate complexes: insights into molecular architectures of BTB-Cul3 ubiquitin ligases. Mol Cell.

[147] Su S, Chen J, Wang Y, Wong LM, Zhu Z, Jiang G, Liu P (2021). Lenalidomide downregulates ACE2 protein abundance to alleviate infection by SARS-CoV-2 spike protein conditioned pseudoviruses. Signal Transduct Target Ther.

[148] Trask AJ, Groban L, Westwood BM, Varagic J, Ganten D, Gallagher PE, Chappell MC, Ferrario CM (2010). Inhibition of angiotensin-converting enzyme 2 exacerbates cardiac hypertrophy and fibrosis in Ren-2 hypertensive rats. Am J Hypertens.

[149] Prasad V, Cerikan B, Stahl Y, Kopp K, Magg V, Acosta-Rivero N, Kim H, Klein K, Funaya C, Haselmann U (2023). Enhanced SARS-CoV-2 entry via UPR-dependent AMPK-related kinase NUAK2. Mol Cell.

[150] Diener K, Wang XS, Chen C, Meyer CF, Keesler G, Zukowski M, Tan TH, Yao Z (1997). Activation of the c-Jun N-terminal kinase pathway by a novel protein kinase related to human germinal center kinase. Proc Natl Acad Sci USA.

[151] Chuang HC, Wang X, Tan TH (2016). MAP4K family kinases in immunity and inflammation. Adv Immunol.

[152] Chuang HC, Lan JL, Chen DY, Yang CY, Chen YM, Li JP, Huang CY, Liu PE, Wang X, Tan TH (2011). The kinase GLK controls autoimmunity and NF-κB signaling by activating the kinase PKC-θ in T cells. Nat Immunol.

[153] Chuang HC, Tsai CY, Hsueh CH, Tan TH (2018). GLK-IKKβ signaling induces dimerization and translocation of the AhR-RORγt complex in IL-17A induction and autoimmune disease. Sci Adv.

[154] Chuang HC, Chen YM, Chen MH, Hung WT, Yang HY, Tseng YH, Tan TH (2019). AhR-ROR-gammat complex is a therapeutic target for MAP4K3/GLK^high^IL-17A^high^ subpopulation of systemic lupus erythematosus. FASEB J.

[155] Hsu CP, Chuang HC, Lee MC, Tsou HH, Lee LW, Li JP, Tan TH (2016). GLK/MAP4K3 overexpression associates with recurrence risk for non-small cell lung cancer. Oncotarget.

[156] Chuang HC, Chang CC, Teng CF, Hsueh CH, Chiu LL, Hsu PM, Lee MC, Hsu CP, Chen YR, Liu YC (2019). MAP4K3/GLK promotes lung cancer metastasis by phosphorylating and activating IQGAP1. Cancer Res.

[157] Chuang HC, Tan TH (2019). MAP4K3/GLK in autoimmune disease, cancer and aging. J Biomed Sci.

[158] Saad MH, Badierah R, Redwan EM, El-Fakharany EM (2021). A comprehensive insight into the role of exosomes in viral infection: dual faces bearing different functions. Pharmaceutics.

[159] Doyle LM, Wang MZ (2019). Overview of extracellular vesicles, their origin, composition, purpose, and methods for exosome isolation and analysis. Cells.

[160] Berenguer J, Lagerweij T, Zhao XW, Dusoswa S, van der Stoop P, Westerman B, de Gooijer MC, Zoetemelk M, Zomer A, Crommentuijn MHW (2018). Glycosylated extracellular vesicles released by glioblastoma cells are decorated by CCL18 allowing for cellular uptake via chemokine receptor CCR8. J Extracell Vesicles.

[161] Cerezo-Magana M, Bang-Rudenstam A, Belting M (2023). Proteoglycans: a common portal for SARS-CoV-2 and extracellular vesicle uptake. Am J Physiol Cell Physiol.

[162] Yeung ML, Teng JLL, Jia L, Zhang C, Huang C, Cai JP, Zhou R, Chan KH, Zhao H, Zhu L (2021). Soluble ACE2-mediated cell entry of SARS-CoV-2 via interaction with proteins related to the renin-angiotensin system. Cell.

[163] Cocozza F, Nevo N, Piovesana E, Lahaye X, Buchrieser J, Schwartz O, Manel N, Tkach M, Thery C, Martin-Jaular L (2020). Extracellular vesicles containing ACE2 efficiently prevent infection by SARS-CoV-2 Spike protein-containing virus. J Extracell Vesicles.

[164] Komander D, Clague MJ, Urbe S (2009). Breaking the chains: structure and function of the deubiquitinases. Nat Rev Mol Cell Biol.

[165] Mettlen M, Chen PH, Srinivasan S, Danuser G, Schmid SL (2018). Regulation of clathrin-mediated endocytosis. Annu Rev Biochem.

[166] Lu Y, Zhu Q, Fox DM, Gao C, Stanley SA, Luo K (2022). SARS-CoV-2 down-regulates ACE2 through lysosomal degradation. Mol Biol Cell.

[167] Vertegaal ACO (2022). Signalling mechanisms and cellular functions of SUMO. Nat Rev Mol Cell Biol.

[168] Seeler JS, Dejean A (2017). SUMO and the robustness of cancer. Nat Rev Cancer.

[169] Jin S, He X, Ma L, Zhuang Z, Wang Y, Lin M, Cai S, Wei L, Wang Z, Zhao Z (2022). Suppression of ACE2 SUMOylation protects against SARS-CoV-2 infection through TOLLIP-mediated selective autophagy. Nat Commun.

[170] Li Z, Yong H, Wang W, Gao Y, Wang P, Chen X, Lu J, Zheng J, Bai J (2023). GSK3326595 is a promising drug to prevent SARS-CoV-2 Omicron and other variants infection by inhibiting ACE2-R671 di-methylation. J Med Virol.

[171] Laine RA (1994). A calculation of all possible oligosaccharide isomers both branched and linear yields 1.05 × 10(12) structures for a reducing hexasaccharide: the Isomer Barrier to development of single-method saccharide sequencing or synthesis systems. Glycobiology.

[172] Reily C, Stewart TJ, Renfrow MB, Novak J (2019). Glycosylation in health and disease. Nat Rev Nephrol.

[173] Mehdipour AR, Hummer G (2021). Dual nature of human ACE2 glycosylation in binding to SARS-CoV-2 spike. Proc Natl Acad Sci USA.

[174] Capraz T, Kienzl NF, Laurent E, Perthold JW, Foderl-Hobenreich E, Grunwald-Gruber C, Maresch D, Monteil V, Niederhofer J, Wirnsberger G (2021). Structure-guided glyco-engineering of ACE2 for improved potency as soluble SARS-CoV-2 decoy receptor. Elife.

[175] Zhao P, Praissman JL, Grant OC, Cai Y, Xiao T, Rosenbalm KE, Aoki K, Kellman BP, Bridger R, Barouch DH (2020). Virus-receptor interactions of glycosylated SARS-CoV-2 spike and human ACE2 receptor. Cell Host Microbe.

[176] Isobe A, Arai Y, Kuroda D, Okumura N, Ono T, Ushiba S, Nakakita SI, Daidoji T, Suzuki Y, Nakaya T (2022). ACE2 N-glycosylation modulates interactions with SARS-CoV-2 spike protein in a site-specific manner. Commun Biol.

[177] Acharya A, Lynch DL, Pavlova A, Pang YT, Gumbart JC (2021). ACE2 glycans preferentially interact with SARS-CoV-2 over SARS-CoV. Chem Commun.

[178] Cao W, Dong C, Kim S, Hou D, Tai W, Du L, Im W, Zhang XF (2021). Biomechanical characterization of SARS-CoV-2 spike RBD and human ACE2 protein-protein interaction. Biophys J.

[179] Chan KK, Dorosky D, Sharma P, Abbasi SA, Dye JM, Kranz DM, Herbert AS, Procko E (2020). Engineering human ACE2 to optimize binding to the spike protein of SARS coronavirus 2. Science.

[180] Jocher G, Grass V, Tschirner SK, Riepler L, Breimann S, Kaya T, Oelsner M, Hamad MS, Hofmann LI, Blobel CP (2022). ADAM10 and ADAM17 promote SARS-CoV-2 cell entry and spike protein-mediated lung cell fusion. EMBO Rep.

